# Supplementary material for: A compassion-based program to reduce psychological distress in medical students: A pilot randomized clinical trial
Source: PLoS One. 2023 Jun 23;18(6):e0287388. doi: 10.1371/journal.pone.0287388 (PMC10289411; doi:10.1371/journal.pone.0287388)
Supplement: S1 File — (DOCX) [file pone.0287388.s002.docx]

**SUPPLEMENTARY MATERIALS**

**A compassion-based program to reduce psychological distress in medical students: a pilot randomized clinical trial**

**Measures**

***Primary outcomes***

*Changes in Compassion to others:* Assessed by Compassion Scale Pommier (CSP), (1) a 24-item self- reported scale rated on a five-point Likert scale *(*from 1= “almost never” to 5= “almost always”). For this study, we used the total score, where higher scores indicate greater compassion to others. Cronbach α for the CSP in this study was .83.

*Changes in Self-compassion:* Assessed by Self-Compassion Scale, Short Form (SCS-SF) (2), a 12-item self-reported scale consisting of three main factors: self-kindness, common humanity, and mindful self-compassion. Each item is rated on a five-point Likert scale (from 1= “almost never” to 5= “almost always”). Higher scores indicate greater self-compassion. Cronbach α for de SCS-SF in this study was .90.

*Changes in Empathy:* Assessed by Interpersonal Reactivity Index (IRI) (3), a 28-item self-report scale measuring empathy towards others. In this study, we only used the Empathic Concern subscale.  Each item is rated on a five-point Likert scale (from 1= “It doesn’t describe me well” to 5= “it describes me well”). Higher scores indicate higher levels of empathy. Cronbach α for the IRI in this study was .76.

*Changes in Psychological distress:* Assessed by Depression Anxiety Stress Scales (DASS-21) (4), a 21-item self-report scale assessing 3 factors: symptoms of depression, anxiety, and stress. Each item is rated on a three-point Likert scale (from 0= “not applicable” to 3= “very applicable to me”). Higher scores indicate more severe symptoms. Cronbach α for the DASS total score in this study was .85.

*Changes in General well-being:* Assessed by Pemberton Happiness Index (PHI) (5), an 11-item scale measuring psychological well-being. In this study, only the remembered well-being scale was included. Each item is rated on a ten-point scale (form 1= “totally disagree” to 10= “totally agree”). Higher scores indicate more happiness. Cronbach α for the PHI in this study was .95.

***Secondary outcomes***

*Changes in Mindfulness:* Assessed by Five Facet Mindfulness Questionnaire – Short Form (FFMQ-SF) (6), a 20-item self-report scale assessing 5 mindfulness facets: observing, describing, acting with awareness, non-judgement of inner experience, and non-reactivity to inner experience. Each item is rated on a five-point Likert scale (from 1= “almost never” to 5= “almost always”). Higher scores indicate greater mindfulness. Cronbach α for the FFMQ-SF total score in this study was .83.

*Changes in Burnout:* Assessed by Maslach Burnout Inventory-Student Survey (MBI-SS) (7), a 15-item self-report scale including 3 factors: emotional exhaustion, cynicism, and academic effectiveness. All items are scored on a 7-point frequency rating scale, ranging from 0 (never) to 6 (every day). Higher scores indicate higher levels of burnout. Cronbach α total score for the MBI-SS in this study was .75.

*Changes in Emotion Regulation:* Assessed by the Difficulties in Emotion Regulation Scale (DERS), (8) a 28 item self-report scale assessing in 6 types of emotion regulation difficulties: emotional inattention, emotional confusion, emotional rejection, emotional lack of control, and emotional life interference. The items are scored on a five-point Likert scale (from 1= “almost never” to 5= “almost always”). Higher scores indicate more difficulties in emotion regulation. Cronbach α for the DERS in this study was .95.

*Changes in Resilience:* Assessed by Brief Resilience Scale (BRS) (9), a 5-item self-reported measure of resilience, ranging from 1 (strongly disagree) to 5 (strongly agree). A higher score indicates a higher resilience. Cronbach α for the BRS in this study was .86.

*Changes in concern about COVID-19:* Assessed by a single-item to measure changes in preoccupation about COVID-19 pandemic.

*Changes in Adherence to the program:* In order to assess the program adherence and program satisfaction, we developed our own measure, adapting the MBI-TAC (10) questionnaire to the CCT program.

**Supplementary Table 1**. CONSORT checklist for pilot trials.

| Section/Topic | Item No | Checklist item | Reported on page No |
| --- | --- | --- | --- |
| Title and abstract | | | |
|  | 1a | Identification as a pilot or feasibility randomised trial in the title | 1 |
|  | 1b | Structured summary of pilot trial design, methods, results, and conclusions (for specific guidance see CONSORT abstract extension for pilot trials) | 2 |
| Introduction | | | |
| Background and objectives | 2a | Scientific background and explanation of rationale for future definitive trial, and reasons for randomised pilot trial | 3-4 |
|  | 2b | Specific objectives or research questions for pilot trial | 5. |
| Methods | | | |
| Trial design | 3a | Description of pilot trial design (such as parallel, factorial) including allocation ratio | 5 |
|  | 3b | Important changes to methods after pilot trial commencement (such as eligibility criteria), with reasons | NA |
| Participants | 4a | Eligibility criteria for participants | 6 |
|  | 4b | Settings and locations where the data were collected | 7 |
|  | 4c | How participants were identified and consented | 6 |
| Interventions | 5 | The interventions for each group with sufficient details to allow replication, including how and when they were actually administered | 7 |
| Outcomes | 6a | Completely defined prespecified assessments or measurements to address each pilot trial objective specified in 2b, including how and when they were assessed | 9 |
|  | 6b | Any changes to pilot trial assessments or measurements after the pilot trial commenced, with reasons | NA |
|  | 6c | If applicable, prespecified criteria used to judge whether, or how, to proceed with future definitive trial | 21 |
| Sample size | 7a | Rationale for numbers in the pilot trial | 5 |
|  | 7b | When applicable, explanation of any interim analyses and stopping guidelines | NA |
| Randomisation: |  |  |  |
| Sequence  generation | 8a | Method used to generate the random allocation sequence | 5 |
|  | 8b | Type of randomisation(s); details of any restriction (such as blocking and block size) | 5 |
| Allocation  concealment  mechanism | 9 | Mechanism used to implement the random allocation sequence (such as sequentially numbered containers), describing any steps taken to conceal the sequence until interventions were assigned | 5 |
| Implementation | 10 | Who generated the random allocation sequence, who enrolled participants, and who assigned participants to interventions | 5 |
| Blinding | 11a | If done, who was blinded after assignment to interventions (for example, participants, care providers, those assessing outcomes) and how | 5 |
|  | 11b | If relevant, description of the similarity of interventions | NA |
| Statistical methods | 12 | Methods used to address each pilot trial objective whether qualitative or quantitative | 10 |
| Results | | | |
| Participant flow (a diagram is strongly recommended) | 13a | For each group, the numbers of participants who were approached and/or assessed for eligibility, randomly assigned, received intended treatment, and were assessed for each objective | 6 |
|  | 13b | For each group, losses and exclusions after randomisation, together with reasons | 6 |
| Recruitment | 14a | Dates defining the periods of recruitment and follow-up | 6 |
|  | 14b | Why the pilot trial ended or was stopped | NA |
| Baseline data | 15 | A table showing baseline demographic and clinical characteristics for each group | 6 |
| Numbers analysed | 16 | For each objective, number of participants (denominator) included in each analysis. If relevant, these numbers  should be by randomised group | 6 |
| Outcomes and estimation | 17 | For each objective, results including expressions of uncertainty (such as 95% confidence interval) for any  estimates. If relevant, these results should be by randomised group | 14 |
| Ancillary analyses | 18 | Results of any other analyses performed that could be used to inform the future definitive trial | 15 |
| Harms | 19 | All important harms or unintended effects in each group (for specific guidance see CONSORT for harms) | 15 |
|  | 19a | If relevant, other important unintended consequences | NA |
| Discussion | | | |
| Limitations | 20 | Pilot trial limitations, addressing sources of potential bias and remaining uncertainty about feasibility | 19 |
| Generalisability | 21 | Generalisability (applicability) of pilot trial methods and findings to future definitive trial and other studies | 20 |
| Interpretation | 22 | Interpretation consistent with pilot trial objectives and findings, balancing potential benefits and harms, and  considering other relevant evidence | 20 |
|  | 22a | Implications for progression from pilot to future definitive trial, including any proposed amendments | 20 |
| Other information | | |  |
| Registration | 23 | Registration number for pilot trial and name of trial registry | 6 |
| Protocol | 24 | Where the pilot trial protocol can be accessed, if available | 6 |
| Funding | 25 | Sources of funding and other support (such as supply of drugs), role of funders | 22 |
|  | 26 | Ethical approval or approval by research review committee, confirmed with reference number | 6 |

**Results**

*Supplementary Figure 1: Effects of CCT program*

*
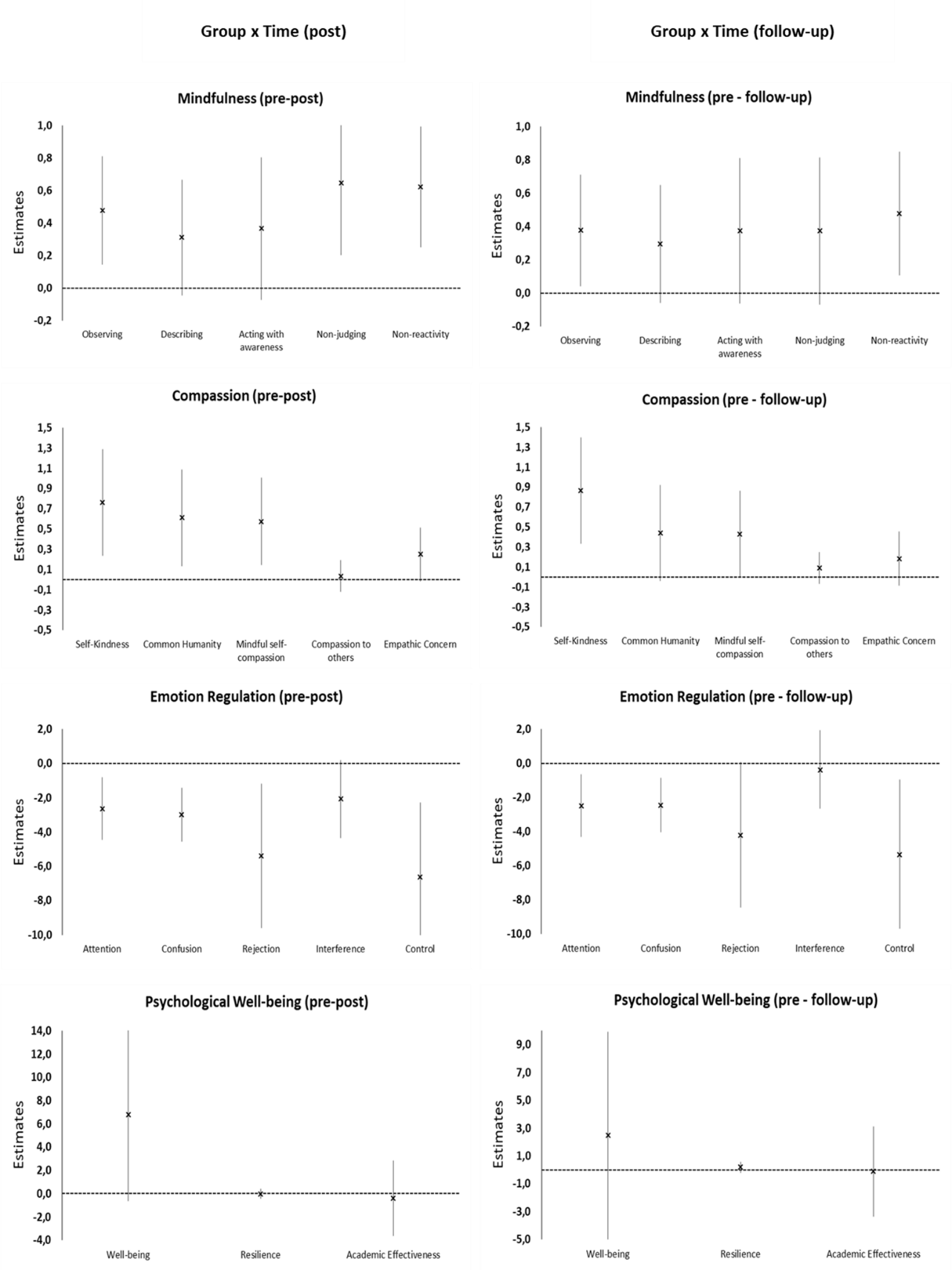
*

Group x Time fixed-effect estimates and their corresponding 95% confidence intervals for each dependent variable. Estimates with CI containing 0 indicate a non-significant interaction. Mixed-effects models analysis.

**Supplementary References**

1. Pommier E, Neff KD, Tóth-Király I. The Development and Validation of the Compassion Scale. Assessment. 2020 Jan 1;27(1):21–39.

2. Raes F, Pommier E, Neff KD, Van Gucht D. Construction and factorial validation of a short form of the Self-Compassion Scale. Clin Psychol Psychother. 2011 May 1;18(3):250–5.

3. Davis MH. A multidimensional approach to individual differences in empathy. https://www.uv.es/friasnav/Davis_1980. 1980. p. 85.

4. Lovibond PF, Lovibond SH. The structure of negative emotional states: Comparison of the Depression Anxiety Stress Scales (DASS) with the Beck Depression and Anxiety Inventories. Behav Res Ther. 1995;33(3):335–43.

5. Hervás G, Vázquez C. Construction and validation of a measure of integrative well-being in seven languages: The Pemberton Happiness Index. Health Qual Life Outcomes. 2013;11(1):66.

6. Baer RA, Smith GT, Hopkins J, Krietemeyer J, Toney L. Using self-report assessment methods to explore facets of mindfulness. Assessment. 2006 Mar;13(1):27–45.

7. Galán F, Sanmartín A, Polo J, Giner L. Burnout risk in medical students in Spain using the Maslach Burnout Inventory-Student Survey. Int Arch Occup Environ Health. 2011 Apr 4;84(4):453–9.

8. Gratz KL, Roemer L. Multidimensional assessment of emotion regulation and dysregulation: Development, factor structure, and initial validation of the difficulties in emotion regulation scale. J Psychopathol Behav Assess. 2004;26(1):41–54.

9. Smith BW, Dalen J, Wiggins K, Tooley E, Christopher P, Bernard J. The brief resilience scale: Assessing the ability to bounce back. Int J Behav Med. 2008 Sep;15(3):194–200.

10. Crane RS, Eames C, Kuyken W, Hastings RP, Williams JMG, Bartley T, et al. Development and Validation of the Mindfulness-Based Interventions - Teaching Assessment Criteria (MBI:TAC). Assessment. 2013 Dec 21;20(6):681–8.
