# Supplementary material for: A compassion-based program to reduce psychological distress in medical students: A pilot randomized clinical trial
Source: PLoS One. 2023 Jun 23;18(6):e0287388. doi: 10.1371/journal.pone.0287388 (PMC10289411; doi:10.1371/journal.pone.0287388)
Supplement: S2 File — (PDF) [file pone.0287388.s003.pdf]

We're building a better ClinicalTrials.gov. Check it out and tell us what you think!

Evaluation of the Efficacy and Mechanisms of Change of Compassion Cultivation Training in Medical Students

The safety and scientific validity of this study is the responsibility of the study sponsor and investigators. Listing a study does not mean it has been evaluated by the U.S. Federal Government. Read our disclaimer for details.

ClinicalTrials.gov Identifier: NCT04690452

Recruitment Status : Completed
First Posted : December 30, 2020
Last Update Posted : November 4, 2022

View this study on Beta.ClinicalTrials.gov

Sponsor:
Universidad Complutense de Madrid

Collaborator:
Nirakara Institute

Information provided by (Responsible Party):
Blanca Rojas, Universidad Complutense de Madrid

Study Details | Tabular View | No Results Posted | Disclaimer | How to Read a Study Record

Study Description

Go to

Brief Summary:

The aim of this randomized, waitlist controlled trial is to examine the efficacy of the Compassion Cultivation Training (CCT©) in reducing psychological distress (i.e., stress, anxiety and depression) and burnout symptoms

while improving psychological well-being medical students. The second goal of the study is to examine whether mindfulness and compassion-related variables as well as emotional-cognitive emotional regulation processes mediate the psychological distress and well-being changes.

The effects of the CCT© program will be measured by means of self-report questionnaires involving different domains (mindfulness, compassion, distress, and well-being measures) at different time points (pre-intervention, inter-session assessment, post-intervention, 2-month and 6-month follow-up).

Condition or disease	Intervention/treatment	Phase
Stress<br>Depression<br>Anxiety<br>Burn Out<br>Well-being<br>Resilience<br>MIndfulness<br>Compassion<br>Self-Compassion<br>Empathy	Behavioral: Compassion Cultivation Training	Not Applicable

Detailed Description:

Compassion and empathy are essential components of health care quality. Several studies have found a significant decrease in empathy and compassion levels during medical school and residency (Hojat, 2004; Bellini, 2005; Stephen, 2006; Neumann, 2011). However, compassion training is usually excluded from medical education. Compassion training in medical students can increase their wellness and decreased burnout (Weingartner, 2019), which in turn improves patients clinical outcomes (Kim, 2004; Rakel, 2009; Hojat, 2011; Attar 2012; Del Canale, 2012; Steinhausen, 2014; Trzeciak, 2017; Moss, 2019). Given that compassion can be trained through standardized interventions (Stephen, 2006; Hojat, 2009a; Goetz, 2010; Kelm, 2014) and educational programs (Patel, 2019), it highlight the need to investigate interventions aimed to improving both, provider self-care and patient care.

Hypothesis:

- Compassion Cultivation Training (CCT©) program would improve psychological well-being while reducing psychological distress (stress, anxiety and depression) and burnout symptoms in medical students as compared to a waitlist control group.
- These improvements would be maintained at 2 and 6 months after finishing the program.
- Mindfulness and compassion changes and emotional-cognitive emotional regulation processes would mediate the relationship between the program and the psychological distress and well-being changes.
- Compassion skills after the program will be a protective factor for stress and worry produced by COVID-19 pandemic.

Procedure:

The study will follow a randomised waitlist controlled trial with five assessment moments (i.e., pre, inter-session, post, 2-month and 6-month follow-ups). Participants will be recruited via constitutional email and the informative screens of the Medical School at Complutense University of Madrid. Participants will be randomized to either CCT© group (N=20) or waiting list control group (N=20). The procedure will include an online assessment via Qualtrics software at the different time points, as well as the completion of a "practice diary" as the inter-session assessment one per week the day before each session.

Program description:

The Compassion Cultivation Training (CCT©) is an 8-week evidence-based standardized meditation program designed at Standford University. The CCT© is aimed at cultivating compassion and empathy toward oneself and others. The program is conducted in groups of 15-20 participants and consisting of weekly 2 hour on-line sessions (due to COVID-19 restrictions) with 20-30 minutes of daily formal meditation practices and informal compassion practices. The CCT© program will be guided by a certified instructor from the Compassion Institute. Program adherence and fidelity will be monitored through revisions of the recorded sessions. The CCT© program comprises six sequential steps: 1) Settling the mind; 2) Loving-kindness and compassion for a loved one; 3) Self-directed loving-kindness and compassion; 4) Common humanity; 5) Cultivating compassion for others; and 6) Active compassion (Tonglen).

Study Design

Go to

Study Type :
Interventional (Clinical Trial)

Actual Enrollment :
40 participants

Allocation:
Randomized

Intervention Model:
Parallel Assignment

Intervention Model Description:
Medical students during academic year 2020-21 will be asked to participate in the present study. Once inclusion criteria are met, each participant will be randomly assigned to the experimental group or waitlist control. Randomization will be performed by using a computer-generated sequence

Masking:
Single (Outcomes Assessor)

Masking Description:
Single

Primary Purpose:
Other

Official Title:
Evaluation of the Efficacy and Mechanisms of Change of Compassion Cultivation Training in Medical Students

Actual Study Start Date :
December 11, 2020

Actual Primary Completion Date :
March 8, 2021

Actual Study Completion Date :
September 24, 2021

Arms and Interventions

Go to

Arm	Intervention/treatment
Experimental: Intervention condition<br>Intervention group that receive a standardized 8 weeks Compassion Cultivation Training from a faculty member certified CCT© instructor (https://www.compassioninstitute.com/about-us/teacher-directory/)	Behavioral: Compassion Cultivation Training<br>The Compassion Cultivation Training (CCT©) is an 8-week standardized meditation program conducted in groups of 15-20 participants and consisting of weekly 2 hour on-line sessions with 20-30 minutes of daily formal meditation practices and informal compassion practices. The CCT© program will be guided by a certified instructor form the Center for Compassion and Altruism Research and Education at Stanford University. The CCT© program comprises six sequential steps: 1) Settling the mind; 2) Loving-kindness and compassion for a loved one; 3) Self-directed loving-kindness and compassion; 4) Common humanity; 5) Cultivating compassion for others; and 6) Active compassion (Tonglen).
Waitlist control condition<br>The participants assigned to the waitlist control will fill in the same questionnaires as the intervention group at the different time points (i.e., pre, post, 2-month and 6-month follow-ups).<br>Two months after finishing the intervention, will become participants of a CCT© program themselves given by the same faculty member certified CCT© teacher as for the experimental group.	Behavioral: Compassion Cultivation Training<br>The Compassion Cultivation Training (CCT©) is an 8-week standardized meditation program conducted in groups of 15-20 participants and consisting of weekly 2 hour on-line sessions with 20-30 minutes of daily formal meditation practices and informal compassion practices. The CCT© program will be guided by a certified instructor form the Center for Compassion and Altruism Research and Education at Stanford University. The CCT© program comprises six sequential steps: 1) Settling the mind; 2) Loving-kindness and compassion for a loved one; 3) Self-directed loving-kindness and compassion; 4) Common humanity; 5) Cultivating compassion for others; and 6) Active compassion (Tonglen).

Outcome Measures

Go to

Primary Outcome Measures :

1. Change in Compassion- Compassion to others at pre, post-intervention and at 2 and 6 months [Time Frame: up to 6 months.]

   Compassion Scale Pommier (CSP; Pommier et al., 2020).

2. Change in Compassion- Self-compassion at pre, post-intervention and at 2 and 6 months [Time Frame: up to 6 months.]

   Self- Compassion Scale (SCS-SF; Raes et al., 2011).

3. Change in Empathy at pre, post-intervention and at 2 and 6 months [Time Frame: up to 6 months.]

   Interpersonal Reactivity Index (IRI, Davis, 1980).

4. Change in Psychological distress- feelings of stress, anxiety and depression at pre, post-intervention and at 2 and 6 months [Time Frame: up to 6 months.]

   Depression Anxiety Stress Scales (DASS- 21; Lovibond, & Lovibond, 1995).

5. Change in General well-being at pre, post-intervention and at 2 and 6 months [Time Frame: up to 6 months.]

   Pemberton Happiness Index (PHI, Hervas, & Vazquez, 2013).

Secondary Outcome Measures :

1. Change in Mindfulness at pre, post-intervention and at 2 and 6 months [Time Frame: up to 6 months.]

   Five Facet Mindfulness Questionnaire (FFMQ; Baer et al., 2006).

2. Change in Burnout at pre, post-intervention and at 2 and 6 months [Time Frame: up to 6 months.]

   Maslach Burnout Inventory (Maslach & Jackson, 1981)

3. Change in Resilience at pre, post-intervention and at 2 and 6 months [Time Frame: up to 6 months.]

   Brief Resilience Scale (BRS; Smith et al., 2008).

4. Change in Emotion regulation at pre, post-intervention and at 2 and 6 months [Time Frame: up to 6 months.]

   Difficulties in Emotion Regulation Scale (DERS, Gratz & Roemer, 2004).

5. Change in Adherence to the program [Time Frame: up to 8 weeks]

   Single-item to measure daily formal meditation practices and informal compassion practices.

6. Changes in State measures during intervention [Time Frame: up to 8 weeks]

   Single-item to measure state changes in: compassion, mindfulness, psychological distress, well-being, and cognitive-emotional regulation processes

Eligibility Criteria

Go to

Information from the National Library of Medicine

Choosing to participate in a study is an important personal decision. Talk with your doctor and family members or friends about deciding to join a study. To learn more about this study, you or your doctor may contact the study research staff using the contacts provided below. For general information, Learn About Clinical Studies.

Ages Eligible for Study:
18 Years and older (Adult, Older Adult)

Sexes Eligible for Study:
All

Accepts Healthy Volunteers:
Yes

Criteria

Inclusion Criteria:

- 18 years of age or more.
- Medical students at University Complutense of Madrid.
- Fluency in oral Spanish
- Providing written, informed consent
- Attendance commitment to all sessions of the program
- Internet and computer access

Exclusion Criteria:

- Severe mental disorder in active phase.
- Being under alcohol and other drugs influence during weekly sessions and assessments

- Participation in another meditation standardized program during CCT©.

Contacts and Locations

Go to

Information from the National Library of Medicine

To learn more about this study, you or your doctor may contact the study research staff using the contact information provided by the sponsor.

Please refer to this study by its ClinicalTrials.gov identifier (NCT number): ***NCT04690452***

Locations

Spain

Universidad Complutense Madrid
Madrid, Spain, 28040

Sponsors and Collaborators

Universidad Complutense de Madrid

Nirakara Institute

Investigators

Principal Investigator: Blanca Rojas, M.D., PhD Universidad Complutense Madrid

More Information

Go to

Additional Information:

The Center for Compassion and Altruism Research and Education EXIT

Compassion Institute EXIT

Center for Mindful Self-Compassion EXIT

The Compassionate Mind Foundation EXIT

Publications:

Hojat M, Mangione S, Nasca TJ, Rattner S, Erdmann JB, Gonnella JS, Magee M. An empirical study of decline in empathy in medical school. Med Educ. 2004 Sep;38(9):934-41. doi: 10.1111/j.1365-2929.2004.01911.x.

Bellini LM, Shea JA. Mood change and empathy decline persist during three years of internal medicine training. Acad Med. 2005 Feb;80(2):164-7. doi: 10.1097/00001888-200502000-00013.

Stepien KA, Baernstein A. Educating for empathy. A review. J Gen Intern Med. 2006 May;21(5):524-30. doi: 10.1111/j.1525-1497.2006.00443.x.

van Donselaar CA, Habbema JD. Recurrence after first seizure. Lancet. 1991 Jan 5;337(8732):46. doi: 10.1016/0140-6736(91)93361-c. No abstract available.

Weingartner LA, Sawning S, Shaw MA, Klein JB. Compassion cultivation training promotes medical student wellness and enhanced clinical care. BMC Med Educ. 2019 May 10;19(1):139. doi: 10.1186/s12909-019-1546-6.

Kim SS, Kaplowitz S, Johnston MV. The effects of physician empathy on patient satisfaction and compliance. Eval Health Prof. 2004 Sep;27(3):237-51. doi: 10.1177/0163278704267037.

Rakel DP, Hoeft TJ, Barrett BP, Chewning BA, Craig BM, Niu M. Practitioner empathy and the duration of the common cold. Fam Med. 2009 Jul-Aug;41(7):494-501.

Hojat M, Louis DZ, Markham FW, Wender R, Rabinowitz C, Gonnella JS. Physicians' empathy and clinical outcomes for diabetic patients. Acad Med. 2011 Mar;86(3):359-64. doi: 10.1097/ACM.0b013e3182086fe1.

Attar HS, Chandramani S. Impact of physician empathy on migraine disability and migraineur compliance. Ann Indian Acad Neurol. 2012 Aug;15(Suppl 1):S89-94. doi: 10.4103/0972-2327.100025.

Del Canale S, Louis DZ, Maio V, Wang X, Rossi G, Hojat M, Gonnella JS. The relationship between physician empathy and disease complications: an empirical study of primary care physicians and their diabetic patients in Parma, Italy. Acad Med. 2012 Sep;87(9):1243-9. doi: 10.1097/ACM.0b013e3182628fbf.

Steinhausen S, Ommen O, Antoine SL, Koehler T, Pfaff H, Neugebauer E. Short- and long-term subjective medical treatment outcome of trauma surgery patients: the importance of physician empathy. Patient Prefer Adherence. 2014 Sep 18;8:1239-53. doi: 10.2147/PPA.S62925. eCollection 2014.

Moss J, Roberts MB, Shea L, Jones CW, Kilgannon H, Edmondson DE, Trzeciak S, Roberts BW. Healthcare provider compassion is associated with lower PTSD symptoms among patients with life-threatening medical emergencies: a prospective cohort study. Intensive Care Med. 2019 Jun;45(6):815-822. doi: 10.1007/s00134-019-05601-5. Epub 2019 Mar 25.

Hojat M. Ten approaches for enhancing empathy in health and human services cultures. J Health Hum Serv Adm. 2009 Spring;31(4):412-50.

Cruz A, Buhling M, Seibel K. [Double blind study of migraine therapy with etilefrine pivalate]. Arzneimittelforschung. 1985;35(7):1086-9. German.

Responsible Party:

Blanca Rojas, Assistant Professor Faculty of Medicine, Universidad Complutense de Madrid

ClinicalTrials.gov Identifier:

NCT04690452 History of Changes

Other Study ID Numbers:

BRLUCM2020-1

First Posted:

December 30, 2020 Key Record Dates

Last Update Posted:

November 4, 2022

Last Verified:

November 2022

Individual Participant Data (IPD) Sharing Statement:

Plan to Share IPD:

No

Studies a U.S. FDA-regulated Drug Product:

No

Studies a U.S. FDA-regulated Device Product:

No

Keywords provided by Blanca Rojas, Universidad Complutense de Madrid:

Compassion
Medical Student
Health
Training program
CCT

Additional relevant MeSH terms:

Burnout, Psychological
Behavioral Symptoms
Stress, Psychological
