# Supplementary material for: A compassion-based program to reduce psychological distress in medical students: A pilot randomized clinical trial
Source: PLoS One. 2023 Jun 23;18(6):e0287388. doi: 10.1371/journal.pone.0287388 (PMC10289411; doi:10.1371/journal.pone.0287388)

Ethics committee

CEIm Hospital Clínico San Carlos

CERTIFIES

That the CEIm Hospital Clínico San Carlos at the Standing Committee meeting, minutes 12.2/20, has evaluated the response to the clarifications requested prior to the study:

- Title: "EVALUATION OF THE EFFECTIVENESS OF A TRAINING PROGRAM IN THE CULTURE OF COMPASSION IN MEDICAL STUDENTS OF THE COMPLUTENSE UNIVERSITY OF MADRID".
- Promoter Code: BRLUCM2020-1
- Internal Code: 20/742-EC_X
- Promoter: NIRAKARA PROYECTOS S.L
- Researcher: MARÍA BLANCA ROJAS LÓPEZ from the Faculty of Medicine of the Complutense University of Madrid.

Document Type	Version
Protocol	Version 2 of 10 December 2020
Patient Information Sheet	V2_October 2020

In this study:

- The necessary requirements for the suitability of the protocol in relation to the objectives of the study are met and the foreseeable risks and discomfort for the subject are justified.
- The procedure for obtaining informed consent is adequate.
- The capacity of the investigator and the means available are adequate to carry out the study.
- The scope of the foreseen economic compensation does not interfere with the respect for ethical postulates.
- The ethical precepts formulated in the Declaration of Helsinki of the World Medical Association on Ethical Principles for Medical Research Involving Human Subjects and its subsequent revisions, as well as those required by the applicable legal regulations according to the characteristics of the study, are complied with.

Therefore, the Committee reports favorably on the realization of said project.

Which I sign in Madrid, on December 14, 2020.

GARCIA ARENILLAS MARIA DEL MAR - 05250249Q

Firmado digitalmente por GARCIA ARENILLAS MARIA DEL MAR - 05250249Q
Fecha: 2020.12.14 13:19:32 +01'00'

Fdo.: Dra. Mar García Arenillas
Presidenta del CEIm Hospital Clínico San Carlos

1. IDENTIFICATION OF THE PROTOCOL

- **EUDRACT number:**
- **Promoter protocol code:** BRLUCM2020-1
- **Version/Date: 10 December 2020**

2. EC TITLE

Evaluation of the effectiveness of a Training Program in the Cultivation of Compassion in medical students of the Complutense University of Madrid.

3. IDENTIFICATION OF THE PROMOTER

Identity: Nirakara Proyectos, S.L. CIF: B-85741262.
Postal address: Centro Superior de Estudios de Gestión, Campus de Somosaguas, 28223 (Somosaguas, Madrid).
Phone: 918530731.
Email: nirakara@nirakara.org.

4. RELEVANT ASPECTS ABOUT THE FINANCING OF THE STUDY:

This study is the continuation of a Teaching Innovation Project (PID) of the UCM that began last year (PID nº 334, 2019-2020), and that has been granted again this year (PID nº 139, 2020-2021). This year's project does not have its own funding.

The Nirakara Institute, associated with the Extraordinary Chair of "Mindfulness and Cognitive Sciences" of the Complutense University of Madrid, will provide the software required for online assessment (i.e., Qualtrics)

The Lilly Foundation will donate a book to each study participant as a final thank you for their participation in the trial.

5. COORDINATING RESEARCHER OF THE STUDY IN SPAIN. ADDRESS OF YOUR WORKPLACE

Maria Blanca Rojas Lopez
Professor of the Faculty of Medicine
Complutense University of Madrid
Seneca Avenue, 2
University City
28040 MADRID
brojas@med.ucm.es

5.1. Collaborating researchers

Pablo Roca Morales
Graduated in Psychology. Master in General Health Psychology.
PDI in training at the UCM (Harvard-UCM predoctoral fellowship)
pabloroc@ucm.es

Maria Teresa Garcia Anton
Graduated in Optics and Optometry

PhD in Vision Sciences
maitegarantucm@ucm.es

Elena Catalan Fernandez
Sixth year student of Medicine of the UCM
elenacat@ucm.es

6. REFERENCE CEIC
Not applicable

7. CENTRES WHERE THE TEST IS PLANNED (see Annex 1)

Principal Investigator	Center
Maria Blanca Rojas Lopez	Faculty of Medicine UCM

8. JUSTIFICATION AND RELEVANCE OF THE STUDY

8.1 Current situation

8.1.1Compassion and medicine

Compassion, the foundation of medical ethics (Fotaki, 2015), suffers a crisis in health today (Trzeciak, 2017). Offering empathetic and compassionate care benefits:

(i) patients by improving clinical outcomes (Kim, 2004; Rakel, 2009; Hojat, 2011; Attar 2012; Del Canale, 2012; Steinhausen, 2014; Trzeciak, 2017; Moss, 2019) -- Patients are open to treatment when they feel heard by their doctors -- (Zolnierek, 2009), decrease depression and improve their quality of life (Burns 1992; Zachariae, 2003; Neumann, 2007) or reduce anxiety in cancer patients (Fogarty, 1999); (i) health professionals, reducing burnout and improving their well-being after repeated contact with the patient's suffering and; iii) to the health system, reducing economic costs (Epstein, 2005). Despite this evidence, doctors often miss opportunities to be compassionate, limiting their performance to biomedical exploration and explanation (Epstein, 2007).

8.1.2. Discomfort in health professionals

Facing suffering without being able to act according to one's beliefs and values due to hierarchical or institutional pressures can lead to burnout in professionals, increase medical errors (Kelm, 2014), obtain suboptimal results in the evolution of patients (Shanafelt, 2002; McHugh, 2011) or decrease empathy and increase dehumanization (Dzeng, 2016). Compassion fatigue, a type of burnout among caregivers of those who suffer (Figley, 1995), is prevalent in medicine (Van Mol, 2015; Sprang, 2007), reaching epidemic levels among licensed and trainee physicians (West, 2016).

8.1.3. Training in compassion and medicine: effects

Empathy and compassion can be trained and improved (Stepien, 2006; Hojat, 2009a; Goetz, 2010; Kelm, 2014). Although related, empathy and compassion stimulate different brain centers; empathy those of pain (Lamm, 2011) and compassion those of reward (Lamm, 2011; Klimecki, 2014). This last aspect acquires relevance in the medical profession, and helps to explain why the practice of compassion constitutes a protective factor against empathic distress, preventing compassion fatigue. Recent studies manifest brain plasticity and suggest that compassion training activates brain regions associated with positive conditions (Klimecki, 2013; Weng, 2013).

8.1.4. Compassion in medical studies

Some studies demonstrate a decrease in empathy and compassion during medical school and residency (Hojat, 2004; Bellini, 2005; Stepien, 2006; Neumann, 2011), especially in the transition to contact with patients (Hojat, 2009b; Neumann, 2011; Wilson, 2012). Other data indicate that medical students start the career in better mental health conditions than individuals of the same age enrolled in other careers, deducing that medical studies contribute to the deterioration of the mental health of students (Brazeau, 2014).

However, compassion is not a primary goal of career teaching even though the compassion of health professionals can be improved with training (Hojat, 2004; Stepien, 2006; Kelm, 2014).

Institutions such as the American Association of Medical Colleges or the American Medical Association are underscoring the importance of compassionate patient care (AAMC; Kelm, 2014). This belief is supported by recent publications (Patel, 2019) and studies conducted with medical students in which it is concluded that: i) compassion training can be an effective and attractive option to reduce burnout and, at the same time, promote the well-being of students and improve their interactions with patients; and ii) teachers should consider offering these skills to students as it appears to reduce empathic erosion suffered by students more directly than stress management training in isolation (Weingartner, 2019).

8.1.5. Previous experience of the research group in teaching the CCT to medical students.

Theresults found by us in the Teaching Innovation Project of the UCM for the 2019-20 academic year (PID nª 334, precedent of the present study collected in PID nº 139 2020-21) coincide with findings from the literature (Weingartner, 2019). In this preliminary study, our students significantly increased: i) their sense of shared humanity; (ii) all parameters related to mindfulness; iii) the ability to remain in the face of discomfort without distress and; iv) self-friendliness (unpublished data, collected in the memory of PID nª 339 2019-20). In summary, the skills learned helped them reduce the stress associated with their personal and academic lives, and reinforce their personal and patient interactions.

8.2. What the test will bring

- Benefits for the health of the participant, derived from developing greater self-compassion, happiness, joy, empathy and compassion towards the other, as well as greater resilience that helps them reduce the levels of stress and anxiety during the race, the exercise of their profession and their personal life.
- Favor the development of courage to approach, understand and relate to suffering (own and others) without having to move away from it (i.e. prevent empathic distress), and the growth of motivation to alleviate suffering.
- Participants, students and future medical professionals, trained in compassion, enjoying greater personal well-being, will have a greater ability to provide compassionate care to their patients, which will result in providing them with more effective care, better therapeutic outcomes and a better coexistence of these with their disease, with the positive impact that this will have on families.
- Establish the bases in the Faculty of Medicine of the UCM to provide society with more compassionate doctors and therefore, favor the growth of a more humane health, in line with the tendency to promote patient-centered health systems
- Contribute to the Faculty of Medicine of the UCM being an example of humanization of teaching.

8.3. Information on the CST™ programme

The CCT™ protocol, developed at Stanford University by contemplative scholars, clinical psychologists and researchers, and currently sponsored by the Institute of Compassion. CCT™ is a secular program that integrates traditional contemplative practices with contemporary psychology and scientific research on compassion. The CCT™ is an 8-week blended course that combines a 2.30-hour face-to-face session each week with internships during the week. The goal of the CCT™ in medicine is to develop a compassionate mind and heart that provides skills for self-care and fosters compassionate interaction with patients.

Compassion, defined as sensitivity to one's own suffering and that of others, along with a deep commitment to seek to alleviate it, is a basic capacity for care inherent in the human being that allows one to respond to suffering with understanding, patience and kindness, rather than with fear and repulsion. Compassion can be trained (Stepien, 2006; Hojat, 2009a, Goetz, 2010; Kelm, 2014), and through his practice, improve compassion for oneself and extend it to those people who are difficult for us. The CCT™ program is based on developing a genuine compassion that arises from recognizing that the suffering of others is similar to our own and that like us, others also wish to be free from such suffering. From this statement, emerges the empathic concern that sensitizes us to the pain of others. Far from implying pity (it places the one who experiences it in a situation of superiority), condescension, permissiveness, or absence of limits, compassion is related to the courage to face suffering and remain in the face of it, and with the motivation to do something to diminish it.

Bibliography

Stephen, K. A., & Baernstein, A. (2006). Educating for Empathy. J Gen Intern Med, 21(5).524–30. PMID: 16704404; PMC1484804. https://doi.org/10.1111/j.1525-1497.2006.00443.x

Hojat, M. (2009a). Ten approaches for enhancing empathy in health and human services cultures. J Health Hum Serv Adm, 31(4):412-450. PMID: 19385420.

Goetz, J. L., Keltner, D., & Simon-Thomas, E. (2010). Compassion: an evolutionary analysis and empirical review. Psychol Bull. 136(3), 351–374. PMID: 20438142 PMCID: 2864937 doi: 10.1037/a0018807

Kelm, Z., Womer, J., Walter, J. K., & Feudtner, C. (2014). Interventions to cultivate physician empathy: a systematic review. *BMC medical education*, *14*(1), 1-11. PMID: 25315848; PMCID: PMC4201694. https://doi.org/10.1186/1472-6920-14-219

Fotaki M. (2015). Why and how is compassion necessary to provide good quality healthcare? IntJ Health Policy Manag, 4(4), PMID: 25844380 PMCID: PMC4380560199–201. https://doi.org/10.15171/ijhpm.2015.66

Trzeciak, S., Roberts, B. W., & Mazzarelli, A. J. (2017). Compassionomics: Hypothesis and experimental approach. Med hypotheses, 107, 92-97. PMID: 28915973 doi: 10.1016/j.mehy.2017.08.015.

Kim, S. S., Kaplowitz, S., & Johnston, M. V. (2004). The effects of physician empathy on patient satisfaction and compliance. Eval Health Prof, 27(3), 237-251. PMID: 15312283 https://doi.org/10.1177/0163278704267037

Rakel, D. P., Hoeft, T. J., Barrett, B. P., Chewning, B. A., Craig, B. M., & Niu, M. (2009). Practitioner empathy and the duration of the common cold. Fam Med, 41(7), 494–501. PMID: 19582635; PMC2720820.

Hojat, M., Louis, D. Z., Markham, F. W., Wender, R., Rabinowitz, C., & Gonnella, J. S. (2011). Physicians' empathy and clinical outcomes for diabetic patients. Acad Medi, 86(3), 359-364. PMID: 21248604 doi: 10.1097/ACM.0b013e3182086fe1

Attar, H. S., & Chandramani, S. (2012). Impact of physician empathy on migraine disability and migraineur compliance. Ann Indian Acad Neurol, 15(Suppl 1), S89–94. PMID: 23024571; PMCID: PMC3444220 https://doi.org/10.4103/0972-2327.100025.

Del Canale, S., Louis, D. Z., Maio, V., Wang, X., Rossi, G., Hojat, M., & Gonnella, J. S. (2012). The relationship between physician empathy and disease complications: an empirical study of primary care physicians and their diabetic patients in Parma, Italy. Ad Med, 87(9), 1243-1249. PMID: 22836852 doi: 10.1097/ACM.0b013e3182628fbf

Steinhausen, S., Ommen, O., Antoine, S. L., Koehler, T., Pfaff, H., & Neugebauer, E. (2014). Short-and long-term subjective medical treatment outcome of trauma surgery patients: the importance of physician empathy. Patient Prefer Adherence, 8, 1239-53. PMID: 25258518; PMCID: PMC4173813. https://doi.org/10.2147/PPA.S62925

Moss, J., Roberts, M. B., Shea, L., Jones, C. W., Kilgannon, H., Edmondson, D. E., & Roberts, B. W. (2019). Healthcare provider compassion is associated with lower PTSD symptoms among patients with life-threatening medical emergencies: a prospective cohort study. Intensive Care Med, 45(6), 815-822. PMID: 30911803. https://doi.org/10.1007/s00134-019-05601-5

Zolnierek, K. B. H., & DiMatteo, M. R. (2009). Physician communication and patient adherence to treatment: a meta-analysis. Medical care, 47(8), 826-34. PMID: 19584762 doi: 10.1097/MLR.0b013e31819a5acc.

Burns, D. D., & Nolen-Hoeksema, S. (1992). Therapeutic empathy and recovery from depression in cognitive-behavioral therapy: a structural equation model. J Consult Clin Psychol, 60(3), 441–9. PMID: 1619098. doi: 10.1037//0022-006x.60.3.441.

Zachariae, R., Pedersen, C. G., Jensen, A. B., Ehrnrooth, E., Rossen, P. B., & von der Maase, H. (2003). Association of perceived physician communication style with patient satisfaction, distress, cancer-related self-efficacy, and perceived control over the disease. Br J Cancer, 88(5), 658-665. PMID: 12618870; PMCID: PMC237635. https://doi.org/10.1038/sj.bjc.6600798

Neumann, M., Wirtz, M., Bollschweiler, E., Mercer, S. W., Warm, M., Wolf, J., & Pfaff, H. (2007). Determinants and patient-reported long-term outcomes of physician empathy in oncology: a structural equation modelling approach. Patient Educ Couns, 69(1-3), 63-75. PMID: 17851016. https://doi.org/10.1016/j.pec.2007.07.003.

Fogarty, L. A., Curbow, B. A., Wingard, J. R., McDonnell, K., & Somerfield, M. R. (1999). Can 40 seconds of compassion reduce patient anxiety? J Clin Oncol, 17(1), 371-371. PMID: 10458256. https://doi.org/10.1200/JCO.1999.17.1.371

Epstein, R. M., Franks, P., Shields, C. G., Meldrum, S. C., Miller, K. N., Campbell, T. L., & Fiscella, K. (2005). Patient-centered communication and diagnostic testing. Ann Fam Med, 3(5), 415-421. PMID: 16189057 PMCID: PMC1466928. doi: 10.1370/afm.348

Epstein, R. M., Hadee, T., Carroll, J., Meldrum, S. C., Lardner, J., & Shields, C. G. (2007). Could this be something serious?. Reassurance, Uncertainty, and Empathy in Response to Patients' Expressions of WorryJ Gen Intern Med, 22(12), 1731-1739. PMID: 17972141 PMCID: PMC2219845 doi: 10.1007/s11606-007-0416-9

Kelm, Z., Womer, J., Walter, J. K., & Feudtner, C. (2014). Interventions to cultivate physician empathy: a systematic review. BMC Medi Edu, 14, 219. PMID: 25315848 PMCID: PMC4201694https://doi.org/10.1186/1472-6920-14-219

Shanafelt, T. D., Bradley, K. A., Wipf, J. E., & Back, A. L. (2002). Burnout and self-reported patient care in an internal medicine residency program. Ann Intern Med, 136(5), 358–367. PMID: 11874308 https://doi.org/10.7326/0003-4819-136-5-200203050-00008

McHugh, M. D., Kutney-Lee, A., Cimiotti, J. P., Sloane, D. M., & Aiken, L. H. (2011). Nurses' widespread job dissatisfaction, burnout, and frustration with health benefits signal problems for patient care. Health aff (Project Hope), 30(2), 202–210. PMID: 21289340 PMCID: PMC3201822https://doi.org/10.1377/hlthaff.2010.0100

Dzeng E. (2016). Moral Distress Amongst Physician Trainees Regarding Futile Treatments. J Gen Intern Med, 31(8), 830. PMID: 26951284 PMCID: PMC4945553 https://doi.org/10.1007/s11606-016-3648-8

Figley, C. R. (1995). Compassion fatigue: Toward a new understanding of the costs of caring. In B. H. Stamm (Ed.), Secondary traumatic stress: Self-care issues for clinicians, researchers, and educators (p. 3–28). The Sidran Press

van Mol, M. M., Kompanje, E. J., Benoit, D. D., Bakker, J., & Nijkamp, M. D. (2015). The Prevalence of Compassion Fatigue and Burnout among Healthcare Professionals in Intensive Care Units: A Systematic Review. PloS one, 10(8), e0136955. PMID: 26322644 PMCID: PMC4554995 doi: 10.1371/journal.pone.0136955

Sprang, G., Clark, J. J., & Whitt-Woosley, A. (2007). Compassion fatigue, compassion satisfaction, and burnout: Factors impacting a professional's quality of life. Journal of Loss and Trauma, 12(3), 259-280.

West, C. P., Dyrbye, L. N., Erwin, P. J., & Shanafelt, T. D. (2016). Interventions to prevent and reduce physician burnout: a systematic review and meta-analysis. Lancet (London, England), 388(10057), 2272–2281. PMID: 27692469https://doi.org/10.1016/S0140-6736(16)31279-X

Lamm, C., Decety, J., & Singer, T. (2011). Meta-analytic evidence for common and distinct neural networks associated with directly experienced pain and empathy for pain. NeuroImage, 54(3), 2492–2502. PMID: 20946964 doi: 10.1016/j.neuroimage.2010.10.014.

Klimecki, O. M., Leiberg, S., Ricard, M., & Singer, T. (2014). Differential pattern of functional brain plasticity after compassion and empathy training. Soc Cogn Affect Neurosci, 9(6), 873–879. PMID: 23576808; PMCID: PMC4040103. https://doi.org/10.1093/scan/nst060

Weng HY, Fox AS, Shackman AJ, et al. Compassion training alters altruism and neural responses to suffering. Psychol Sci. 2013;24(7):1171-1180. PMID: 23696200 PMCID: PMC3713090 doi:10.1177/0956797612469537

Hojat, M., Mangione, S., Nasca, T. J., Rattner, S., Erdmann, J. B., Gonnella, J. S., & Magee, M. (2004). An empirical study of decline in empathy in medical school. Med Educ, 38(9), 934–941. PMID: 15327674 https://doi.org/10.1111/j.1365-2929.2004.01911.x

Bellini, L. M., & Shea, J. A. (2005). Mood change and empathy decline persist during three years of internal medicine training. Academic medicine: journal of the Association of American Medical Colleges, 80(2), 164–167. https://doi.org/10.1097/00001888-200502000-00013

Neumann, M., Edelhäuser, F., Tauschel, D., Fischer, M. R., Wirtz, M., Woopen, C., Haramati, A., & Scheffer, C. (2011). Empathy decline and its reasons: a systematic review of studies with medical students and residents. Ad Med, 86(8), 996–1009. PMID:1670661 https://doi.org/10.1097/ACM.0b013e318221e615

Hojat, M., Vergare, M. J., Maxwell, K., Brainard, G., Herrine, S. K., Isenberg, G. A., Veloski, J., & Gonnella, J. S. (2009b). The devil is in the third year: a longitudinal study of erosion of empathy in medical school. Ad Med, 84(9), 1182–1191. PMID: 19707055 https://doi.org/10.1097/ACM.0b013e3181b17e

Wilson, S. E., Prescott, J., & Becket, G. (2012). Empathy levels in first- and third-year students in health and non-health disciplines. Am J Pharm Educ, 76(2), 24. PMID: 22438596 PMCID: PMC3305933https://doi.org/10.5688/ajpe76224PMID: 22438596 PMCID: PMC3305933 doi:10.5688/ajpe76224

Brazeau, C. M., Shanafelt, T., Durning, S. J., Massie, F. S., Eacker, A., Moutier, C., Satele, D. V., Sloan, J. A., & Dyrbye, L. N. (2014). Distress among matriculating medical students relative to the general population. Acad Med, 89(11), 1520–1525. PMID: 25250752 doi:https://doi.org/10.1097/ACM.0000000000000482

Association of American Medical Colleges (AAMC). Learning objectives for medical student education guidelines for medical schools. https:// members. AAMC. org/ eweb/ upload/ Learning Objectives for Medical Student Educ Report I.pdf. https://www.aamc.org/system/files/c/2/493604-umecurriculumdashboardresource.pdf (accessed 10-7-2020)

Patel, S., Pelletier-Bui, A., Smith, S., Roberts, M. B., Kilgannon, H., Trzeciak, S., & Roberts, B. W. (2019). Curricula for empathy and compassion training in medical education: A systematic review. PloS one, 14(8), e0221412. PMID: 31437225 PMCID: PMC6705835 doi: 10.1371/journal.pone.0221412

Patel, S., Pelletier-Bui, A., Smith, S., Roberts, M. B., Kilgannon, H. J., Trzeciak, S., & Roberts, B. W. (2018). Curricula and methods for physician compassion training: protocol for a systematic review. BMJ open, 8(9), e024320. PMID: 30224405 PMCID: PMC6144316 https://doi.org/10.1136/bmjopen-2018-024320

Weingartner LA, Sawning S, Shaw MA, Klein JB. (2019). Compassion cultivation training promotes medical student wellness and enhanced clinical care. BMC Med Educ, 10;19(1):139. PMID: 31077192; PMCID: PMC6511143. DOI: 10.1186/s12909-019-1546-6

8.4. Hypothesis

1. The standardized Compassion Cultivation Training (CCT) program will produce an improvement in emotional well-being and a reduction in psychological distress (stress, anxiety, depression) and burnout of post-program medical students compared to a control group on the waiting list.

2. Such changes shall be maintained over time two and six months after the end of the programme.

3. The above changes will be driven by increases in compassion for oneself, compassion for others, attention to the present moment, and emotional regulation skills.

4. The skills developed in the program will be a protective factor for the management of stress and psychological distress caused by the COVID-19 pandemic.

9. DESIGN

A randomized, controlled, unicentric trial will be conducted with a control group on the waiting list (parallel groups). A 1:1 randomization will be performed (using the relevant function in Excell), with a single-blind masking. The simple blind is guaranteed by the division of weights in the research team, so that the person in charge of the evaluation and analysis of data does not participate and is blind to the process of randomization and the formation of the groups (RP). The IP of the project (BR) will be in charge of summoning the participants and assigning the groups.

9.1 Study design

We will perform an analysis of the participants' responses to an online protocol made up of different psychometrically validated psychological measures, which will be completed by the participants at different times (Fig. 1). These questionnaires will be used as quantitative indicators of the psychological changes experienced by the participants in the main outcomes and targets of the CCT® program.

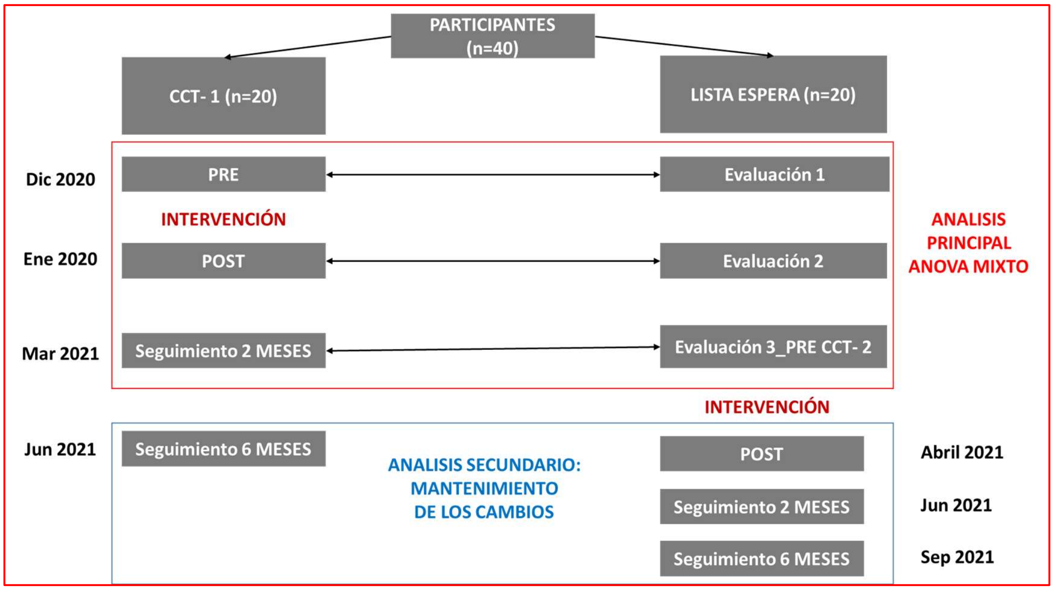

Figure 1. CONSORT diagram

In a **first phase**, participants will be randomized into the two study groups (Fig. 1): experimental group (i.e., CCT program) or control group on waiting list. Such randomization shall be carried out after baseline assessment to avoid the occurrence of biases produced by expectations of participation in the CCT© program.

In a **second phase**, 2 months after the end of the first CST programme (CST-1), in February 2021, participants on the waiting list will be offered the possibility of participating in the CST programme by becoming the CCT-2©© group.

People who voluntarily want to participate in the research will commit to carry out a pre-program© evaluation, a brief inter-sessional evaluations during the program, an evaluation at the end of the program and an evaluation at 2 and 6 months after the end of the program. During the first phase of the study, the waiting list group (control group) will undergo the same evaluations and at the same times as the CCT-1 group.

9.2 Online questionnaires

They will be applied through the online platform "Qualtrics". The technical details of the questionnaires to be completed by the participants are specified below.

9.2.1 Block of pre-post programme questionnaires (Table 1)

Table 1. Questionnaires to be completed by pre-post program participants

Construct	Instrument
	Sociodemographic and health data*
	Previous experience in meditation
	Impact of COVID-19 on Students
	**Mindfulness and compassion**
Mindfulness	Five Facet Mindfulness Questionnaire (FFMQ; Baer et al., 2006) → short version of 20 items
Compassion for oneself	Self-Compassion Scale (SCS-SF; Raes et al., 2011) → Short version of 12 items
Compassion for others	CS Pommier Compassion Scale (CSP; Pommier et al., 2020) 24 items→
Empathy	Interpersonal Reactivity Index (IRI, Davis, 1980) 14 items (subscales of empathic concern and personal discomfort)→
	**Psychopathology**
Stress, anxiety and depression	Depression Anxiety Stress Scales (DASS – 21; Lovibond, & Lovibond, 1995) 21 items.→
Emotional regulation	Difficulties in Emotion Regulation Scale (DERS, Gratz & Roemer, 2004) 28 items→
Burnout	Maslach Burnout Inventory (student version) 15 items→
	**Welfare**
General well-being	Pemberton Happiness Index (PHI, Hervas, & Vazquez, 2013) 11 items→
Resilience	Brief Resilience Scale (BRS; Smith et al., 2008) 5 items→

9.2.2 Block of inter-sessional questionnaires (once a week)

The inter-session evaluations will consist of a brief questionnaire aimed at evaluating adherence to the program and weekly practice. In addition, at the equator and at the end of the program, the participant will complete an anonymous survey required by the Compassion Institute to evaluate the partnership with the program, the alliance with the instructor and the satisfaction with the program. In addition, to ensure and evaluate adherence to the protocol by the instructor, the sessions of the program will be recorded for later review.

10. MAIN OBJECTIVE

Main objective

The objective of this proposal is to evaluate the efficacy and psychological changes produced by a standardized program of Training in the Cultivation of Compassion (CCT©)

in medical students of the Complutense University of Madrid compared to a control group on the waiting list.

Secondary objectives

1) Examine whether the changes produced by the program are maintained two and six months after the end of the program.
2) Examine changes in psychological mechanisms mediating positive program outcomes. To assess whether compassion training is a protective factor against the stress and psychological distress caused by the COVID-19 pandemic.

11. EXPERIMENTAL DRUG AND CONTROL. DOSAGE, FITNESS, VIA

11.1 Choice of CST programme

The CCT© program was developed in 2009 at Stanford University by contemplative scholars, clinical psychologists and researchers, and is currently sponsored by the Compassion Institute. CCT© is a secular program that integrates traditional contemplative practices with contemporary psychology and scientific research on compassion. The CCT© is an 8-week blended course that combines a 2-hour face-to-face session each week with internships during the week. The goal of the CCT© in medicine is to develop a compassionate mind and heart that provides skills for self-care and fosters compassionate interaction with patients.

Compassion, defined as sensitivity to one's own suffering and that of others, along with a deep commitment to seek to alleviate it, is a basic capacity for care inherent in the human being that allows one to respond to suffering with understanding, patience and kindness, rather than with fear and repulsion. Compassion can be trained (Stepien, 2006; Hojat, 2009a, Goetz, 2010; Kelm, 2014), and through his practice, improve compassion for oneself and extend it to those people who are difficult for us. The CCT™ program is based on developing a genuine compassion that arises from recognizing that the suffering of others is similar to our own and that like us, others also wish to be free from such suffering. From this statement, emerges the empathic concern that sensitizes us to the pain of others. Far from implying pity (it places the one who experiences it in a situation of superiority), condescension, permissiveness, or absence of limits, compassion is related to the courage to face suffering and remain in the face of it, and with the motivation to do something to diminish it.

11.2 Program Details

The CCT© program consists of 6 steps that are developed in eight weeks. Specifically:

Week 1. Settlement and approach
Development of mindfulness, settling the mind on the present experience with acceptance and without judgment through exercises aimed at focusing attention on the breath by loving, with kindness, attention back whenever it is distracted.

Week 2. Compassion as a natural capacity
Learn to recognize the physical and psychic signs associated with compassion through dialogue and guided exercises.

Week 3. Self-pity
It presents the idea that the cultivation of self-compassion is the basis for developing compassionate behavior towards others. Training in the practice of self-compassionate dialogue, self-acceptance and growth of these skills in difficult situations.

Week 4. Self-love
Learn to cultivate warmth, appreciation, joy and gratitude for oneself.

Week 5. Shared humanity
Recognition of shared humanity (seeing the other "just as I am") and interdependence as the foundation of compassion toward others.

Week 6. Compassion
From shared humanity and interdependence, cultivate compassion for all beings, including difficult ones.

Week 7. Active compassion
Aimed at developing inner stability to welcome the suffering of others and offer one's own happiness.

Week 8. Integrated Compassion Cultivation Practice
Bring together the elements of the previous steps into a compassionate meditation practice that can be followed as a daily practice

These six steps will be developed through:

- 8 weekly meetings of 2h online duration (due to COVID-19) consisting of relational exercises, meditations and dialogues on pedagogy and science related to the theme of the week. The online meetings will be held through the Zoom platform.
- Guided meditations recorded in audios of 20-30 min duration to be carried out daily (formal practices).
- Informal practices in everyday life

11.3 Procedure for assessing participant compliance

Inter-session surveys together with the number of sessions attended by the participant will be used to assess adherence to the program.

12. PRIMARY ENDPOINT

- Main variables:
  - Psychological distress: stress, anxiety, depression.
  - Psychological well-being.
  - Burnout
  - Compassion
- Secondary variables:
  - Mindfulness or attention to the present.
  - Empathy.
  - Emotional regulation.
  - Resilience.
  - Adherence to the program.
  - Daily practice.

13. STUDY POPULATION AND TOTAL NUMBER OF PATIENTS

13. 1. Sample calculation method

The sample size was determined a priori using the G*Power software (v. 3.0.10). The sample size required to analyze a mixed ANOVA (the group factor as an independent measure and the time factor as a related measure), with an expected effect size of 0.40 (Brito-Pons et al., 2018), a significance level of 0.05, and a power of 90%, the estimated sample size is 44 participants in the entire sample (22 in each group). This estimation of the sample size has been carried out for the pre-post change in the variable of emotional distress (i.e., stress, anxiety and depression).

13.2. Estimation of possible loss

In order to minimize the sample loss throughout the study, we have adopted several measures aimed at maintaining motivation and reinforcing the participation of the participants: the members of the control group will sign an agreement to carry out the relevant evaluations to formalize the reservation of their place in the second promotion of the course. Once the study is completed, participants will be rewarded with an ECTS credit of free configuration (approved by the UCM Studies Commission on November 12, 2020) and object of academic recognition for the degree in medicine. In addition, participants who complete all evaluations of the study will receive a gift book on the theme of the program. For all the above, we expect a sample loss of less than 5%.

13.4. Description of recruitment and requirements of participants

The CCT® program will be offered to all students of the Faculty of Medicine of the UCM enrolled in the 2020-21 academic year. This offer will be made through the Vice-rectorate of students of the Faculty of Medicine of the UCM, the dissemination screens of the library, the association of students of said faculty and course delegates. The selection of the participants in the study will be made among those students who apply for enrollment in the CCT® program. In the selection, preference will be given to those students who are in the 3rd-6th year of training because it is the period in which they are in contact with patients in the different hospitals associated with the UCM.

Study inclusion criteria include: 1) being of legal age; 2) sufficient level of Spanish to understand the instructions and sessions of the program; 3) be enrolled in the Faculty of Medicine of the UCM during the academic year 2020-21; (4) commitment to attend the entire programme; 5) provide informed consent to participate in the study; and 6) have access to the internet and computer to be able to carry out the online sessions.

Exclusion criteria include: (1) having a severe mental disorder in the active phase; 2) be under the influence of alcohol or other substances during the evaluation of the criteria or the sessions of the program; and 3) participate in another standardized meditation program during the course of the program.

The criterion of attendance at 75% of the sessions of the program will be used as an indication of "minimal exposure to treatment".

14. STATISTICAL ANALYSIS

Following the CONSORT recommendations, Intention-To-Treat Analysis will be performed to handle lost data. A diagnosis of the randomness pattern of the loss will begin using Little's MCAR test. The baseline differences between completers (i.e., per-protocol) and missing will also be analyzed to determine if there are any variables in the dataset that predict data loss. Depending on the loss pattern, multiple imputations will be made or by means of Maximum Likelihood algorithms. Finally, sensitivity analyses will be performed comparing the per-protocol data with the imputed ones to determine if the imputation has generated biased estimates. As indicated in the previous section, the criterion of attendance at 75% of the sessions of the program will be used as an indicator of "minimal exposure to treatment".

Student's t-tests and chi-square tests will be used to analyze differences between groups at baseline. The main analysis will be a mixed ANOVA with two factors: independent measures group factor (experimental vs control) and repeated measures time factor (pre, post, follow-up 2 and 6 months). ANCOVAs will also be performed using baseline as a covariate in analyses and repeated measure ANOVAs to assess the maintenance of changes over time. The analysis of variance will be complemented by mediating and moderating analyses to study the mechanisms of change of the program. A significance

level of alpha 0.05 will be defined in bilateral contrasts. Statistical analyses will be carried out using SPSS (v. 25) and R (v. 3.6.1) software.

15. ETHICAL CONSIDERATIONS

Given the nature of the study that is presented for evaluation by the Ethics Committee, the research team will follow at all times during the development of the trial the ethical standards set by the Code of Ethics of the Psychologist published by the Official College of Psychologists (https://www.cop.es/pdf/Codigo-Deontologico-Consejo-Adaptacion-Ley-Omnibus.pdf). Research participants shall not be exposed to any permanent, irreversible or unnecessary harm, and their participation in the research shall be explicitly authorised by informed consent (Art. 34). Likewise, the research will respect the dignity of people, their beliefs, their privacy and their modesty (art. 37). Regarding the use of the information, only the information strictly necessary for the performance of the tasks and always with the express authorization of the participants in the research will be collected (art. 39).

All information obtained will be subject to the duty and right of professional secrecy (art. 40) and will be treated with absolute confidentiality, maintaining the anonymity of the participants in any publication to which the project could give rise. In any case, all the members of this project will adapt to the provisions of Organic Law 3/2018 of December 5 on the Protection of Personal Data and Royal Decree 1720/2007, of December 21, which develops said Law.

The study that is proposed for evaluation by the Committee, supposes a practically non-existent risk for the participant since during the development of the program, he will be repeatedly warned to prioritize his well-being over the recommended practices and exercises as well as to perform them within an emotional safety zone, being encouraged to suspend them and consult the instructor before any incident. The instructor will be available to participants throughout the duration of the study via email, personal telephone or online videoconference to meet the needs of the participants. In addition, during all online sessions and following the recommendations of the Compassion Institute to offer the CCT© program online, and in order to better monitor the participants by the instructor during online connections, he will preferably be assisted by another person in the technical issues of the connection. In this study, this work will be carried out by a sixth-grade medical student member of the Teaching Innovation Project in which this study is framed.

The present clinical trial uses questionnaires and extended measures used in clinical and psychological research (see section 9.2.1) and meditation (mainly attentional and phenomenological aspects). All participants are volunteers and if a high score is observed in the depression or anxiety questionnaires, they will be informed of the availability of assistance services, if they wish, both in the Faculty of Psychology of the UCM and in the field of the public health system. The participation in the project team

of a Bachelor of Medicine and Surgery (María Blanca Rojas López) and a graduate in Psychology (Pablo Roca Morales) guarantee the adequate monitoring of the emotional evolution of the participants. Taking into account the exploratory nature of the study (it is one of the first RCTs randomized applying the CCT program in future medical professionals), we consider that a control group on the waiting list meets the relevant requirements to respond to the hypotheses of the study.

The Nirakara Institute (member of the Extraordinary Chair of "Mindfulness and Cognitive Sciences" of the Complutense University of Madrid) and participant in this clinical trial, rigorously observes the ethical aspects and data handling procedures, complying with the LOPD. This Institute has passed an audit of the Data Protection Agency having its files discharged for "high grade" research purposes. Specifically, the audit has verified and approved the standard of the use of "samples not associated with an identified or identifiable person because the information that identifies that person has been replaced or detached using a code that allows the reverse operation". This procedure is provided for in the present proposal.

The information to the participants will be provided by the person in charge of the Teaching Innovation Project in which this trial is registered and who in turn is the principal investigator of the clinical trial and instructor of the CCT© program.

Monitoring, audits, CEIC reviews and regulatory inspections related to the test will be allowed, facilitating direct access to the original documents/data.

16. DURATION OF TREATMENT (of the stay in the EC in this case)

Participants will formally remain within the clinical trial from November 2020 to June and October 2021 depending on whether they belong to the CCT-1 or CCT-2 group respectively.

17. TIMELINE AND EXPECTED COMPLETION DATE

Start in the first half of November 2020 and end in the second half of September 2021.

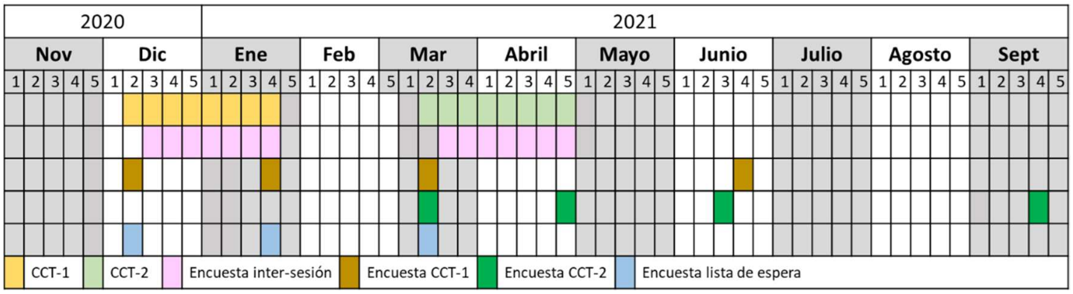

Supplement: S3 File — (PDF) [file pone.0287388.s004.pdf]
