# Supplementary material for: A compassion-based program to reduce psychological distress in medical students: A pilot randomized clinical trial
Source: PLoS One. 2023 Jun 23;18(6):e0287388. doi: 10.1371/journal.pone.0287388 (PMC10289411; doi:10.1371/journal.pone.0287388)

Dictamen Protocolo Favorable

C.P. BRLUCM2020-1
C.I. 20/742-EC_X

14 de diciembre de 2020

CEIm Hospital Clínico San Carlos

Dra. Mar García Arenillas
Presidenta del CEIm Hospital Clínico San Carlos

CERTIFICA

Que el CEIm Hospital Clínico San Carlos en reunión de Comisión Permanente, acta 12.2/20, ha evaluado la respuesta a las aclaraciones solicitadas con anterioridad al estudio:

Título: ***"EVALUACIÓN DE LA EFICACIA DE UN PROGRAMA DE ENTRENAMIENTO EN EL CULTIVO DE LA COMPASIÓN EN ALUMNOS DE MEDICINA DE LA UNIVERSIDAD COMPLUTENSE DE MADRID"***
Código Promotor: BRLUCM2020-1
Código Interno: 20/742-EC_X

Promotor: NIRAKARA PROYECTOS S.L
Investigadora: MARÍA BLANCA ROJAS LÓPEZ de la Facultad de Medicina de la Universidad Complutense de Madrid

Tipo Documento	Versión
Protocolo	Versión 2 de 10 diciembre 2020
Hoja Información de Paciente	V2_Octubre 2020

Que en este estudio:

- Se cumplen los requisitos necesarios de idoneidad del protocolo en relación con los objetivos del estudio y están justificados los riesgos y molestias previsibles para el sujeto.
- Es adecuado el procedimiento para obtener el consentimiento informado.
- La capacidad del investigador y los medios disponibles son adecuados para llevar a cabo el estudio.
- El alcance de las compensaciones económicas previstas no interfiere con el respeto de los postulados éticos.
- Se cumplen los preceptos éticos formulados en la Declaración de Helsinki de la Asociación Médica mundial sobre principios éticos para las investigaciones médicas en seres humanos y en sus posteriores revisiones, así como aquellos exigidos por la normativa legal aplicable en función de las características del estudio.

Es por ello que el Comité **informa favorablemente** sobre la realización de dicho proyecto.

Lo que firmo en Madrid, a 14 de diciembre de 2020

GARCIA ARENILLAS MARIA DEL MAR - 05250249Q
Firmado digitalmente por GARCIA ARENILLAS MARIA DEL MAR - 05250249Q
Fecha: 2020.12.14 13:19:32 +01'00'

Fdo.: Dra. Mar García Arenillas
Presidenta del CEIm Hospital Clínico San Carlos

Hospital Clínico San Carlos
Profesor Martín Lagos, s/n. - Puerta G - 4ª Norte Madrid 28040 Madrid España
Tel. 91 330 38 19 Fax. 91 330 32 99 Correo electrónico ceic.hcsc@salud.madrid.org

1. IDENTIFICACIÓN DEL PROTOCOLO

- **Número EUDRACT:**
- **Código de protocolo del promotor:** BRLUCM2020-1
- **Versión/Fecha: 10 diciembre 2020**

2. TÍTULO DEL EC

Evaluación de la eficacia de un programa de Entrenamiento en el Cultivo de la Compasión en alumnos de medicina de la Universidad Complutense de Madrid.

3. IDENTIFICACIÓN DEL PROMOTOR

Identidad: Nirakara Proyectos, S.L. CIF: B-85741262.
Dirección postal: Centro Superior de Estudios de Gestión, Campus de Somosaguas, 28223 (Somosaguas, Madrid).
Teléfono: 918530731.
Correo electrónico: nirakara@nirakara.org.

4. ASPECTOS RELEVANTES SOBRE LA FINANCIACIÓN DEL ESTUDIO:

El presente estudio es la continuación de un Proyecto de Innovación Docente (PID) de la UCM que comenzó el curso pasado (PID nº 334, 2019-2020), y que ha sido concedido de nuevo este año (PID nº 139, 2020-2021). El proyecto de este año no dispone de financiación propia.

El Instituto Nirakara, asociado a la Cátedra Extraordinaria de «Mindfulness y Ciencias Cognitivas» de la Universidad Complutense de Madrid, proporcionará el software requerido para la evaluación online (i.e., Qualtrics)

La fundación Lilly donará un libro a cada participante en el estudio como agradecimiento final por su participación en el ensayo.

5. INVESTIGADOR COORDINADOR DEL ESTUDIO EN ESPAÑA. DIRECCIÓN DE SU CENTRO DE TRABAJO

María Blanca Rojas López
Profesor Titular de la Facultad de Medicina
Universidad Complutense de Madrid
Avda. de Séneca, 2
Ciudad Universitaria
28040 MADRID
brojas@med.ucm.es

5.1. Investigadores Colaboradores

Pablo Roca Morales
Graduado en Psicología. Máster en Psicología General Sanitaria.
PDI en formación en la UCM (Beca predoctoral Harvard-UCM)
pabloroc@ucm.es

María Teresa García Antón
Graduada en Óptica y Optometría
Dra. en Ciencias de la Visión
maitegarantucm@ucm.es

Elena Catalán Fernández
Estudiante de sexto de Medicina de la UCM
elenacat@ucm.es

6. CEIC DE REFERENCIA
No procede

7. CENTROS DONDE SE PREVÉ REALIZAR EL ENSAYO (ver Anexo 1)

Investigador principal	Centro
María Blanca Rojas López	Facultad de Medicina UCM

8. JUSTIFICACIÓN Y PERTINENCIA DEL ESTUDIO

8.1 Situación actual

8.1.1 Compasión y medicina

La compasión, fundamento de la ética médica (Fotaki, 2015), sufre una crisis en la sanidad actual (Trzeciak, 2017). Ofrecer un cuidado empático y compasivo beneficia:

i) a los pacientes, al mejorar los resultados clínicos (Kim, 2004; Rakel, 2009; Hojat, 2011; Attar 2012; Del Canale, 2012; Steinhausen, 2014; Trzeciak, 2017; Moss, 2019) -- Los pacientes se muestran abiertos al tratamiento cuando se sientes escuchados por sus médicos -- (Zolnierek, 2009), disminuir la depresión y mejorar su calidad de vida (Burns 1992; Zachariae, 2003; Neumann, 2007) o disminuir la ansiedad en pacientes oncológicos (Fogarty, 1999); i) a los profesionales de la salud, reduciendo el burnout y mejorando su bienestar tras el contacto repetido con el sufrimiento del paciente y; iii) al sistema sanitario, disminuyendo los costes económicos (Epstein, 2005). A pesar de estas evidencias, los médicos desaprovechan, con frecuencia, ocasiones de ser compasivos, limitando, su actuación, a la exploración y explicación biomédica (Epstein, 2007).

8.1.2. Malestar en los profesionales de la salud

Enfrentarse al sufrimiento sin poder actuar de acuerdo a las propias creencias y valores debido a presiones jerárquicas o institucionales puede conducir al burnout en los profesionales, aumentar los errores médicos (Kelm, 2014), obtener resultados

subóptimos en la evolución de los pacientes (Shanafelt, 2002; McHugh, 2011) o disminuir la empatía y aumentar la deshumanización (Dzeng, 2016). La fatiga por compasión, tipo de burnout entre cuidadores de los que sufren (Figley, 1995), es prevalente en medicina (Van Mol, 2015; Sprang, 2007), alcanzando niveles epidémicos entre los médicos licenciados y en formación (West, 2016).

8.1.3. Entrenamiento en compasión y medicina: efectos

La empatía y la compasión pueden entrenarse y mejorarse (Stepien, 2006; Hojat, 2009a; Goetz, 2010; Kelm, 2014). Aunque relacionadas, la empatía y la compasión estimulan centros cerebrales diferentes; la empatía los del dolor (Lamm, 2011) y la compasión los de recompensa (Lamm, 2011; Klimecki, 2014). Este último aspecto adquiere relevancia en la profesión médica, y contribuye a explicar porque la práctica de compasión constituye un factor protector del distrés empático, previniendo la fatiga por compasión. Estudios recientes manifiestan la plasticidad del cerebro y apuntan que el entrenamiento en compasión activa regiones del cerebro asociadas con afecciones positivas (Klimecki, 2013; Weng, 2013).

8.1.4. Compasión en los estudios de medicina

Algunos estudios demuestran una diminución de la empatía y la compasión durante la carrera de medicina y la residencia (Hojat, 2004; Bellini, 2005; Stepien, 2006; Neumann, 2011), especialmente, en la transición al contacto con los pacientes (Hojat, 2009b; Neumann, 2011; Wilson, 2012). Otros datos apuntan que los estudiantes de medicina comienzan la carrera en mejores condiciones de salud mental que individuos de la misma edad inscritos en otras carreras, deduciéndose que los estudios de medicina contribuyen al deterioro de la salud mental de los estudiantes (Brazeau, 2014).

No obstante, la compasión no es un objetivo primario de enseñanza en la carrera a pesar de que la compasión de los profesionales de la salud puede mejorar con entrenamiento (Hojat, 2004; Stepien, 2006; Kelm, 2014).

Instituciones como la Asociación Americana de Universidades de Medicina o la Asociación Americana de Medicina, están subrayando la importancia de ofrecer un cuidado compasivo al paciente (AAMC; Kelm, 2014). Esta creencia se ve respaldada por publicaciones recientes (Patel, 2019) y estudios llevados a cabo con estudiantes de medicina en los que se concluyendo que: i) un entrenamiento en compasión puede ser una opción eficaz y atractiva para reducir el burnout y, al mismo tiempo, promover el bienestar de los estudiantes y mejorar sus interacciones con los pacientes; y ii) el profesorado debería considerar ofrecer estas habilidades a los estudiante ya que parece reducir la erosión empática que sufren los estudiantes de una forma más directa que el entrenamiento en el manejo del estrés de forma aislada (Weingartner, 2019).

8.1.5. Experiencia previa del grupo de investigación en le enseñanza del CCT en estudiantes de medicina.

Los resultados encontrados por nosotros en el Proyecto de Innovación Docente de la UCM para el curso 2019-20 (PID nª 334, precedente del presente estudio recogido en el PID nº 139 2020-21) coinciden con hallazgos de la literatura (Weingartner, 2019). En este estudio preliminar, nuestros estudiantes incrementaron significativamente: i) su sentimiento de humanidad compartida; ii) todos los parámetros relacionados con la atención plena; iii) la capacidad de permanecer ante el malestar sin distrés y; iv) la amabilidad hacia uno mismo (datos no publicados, recogidos en la memoria del PID nª 339 2019-20). En resumen, las habilidades aprendidas les ayudaron a disminuir el estrés asociado a su vida personal y académica, y a reforzar sus interacciones personales y con los pacientes.

8.2. Qué aportará el ensayo

- Beneficios para la salud del participante, derivados de desarrollar mayor autocompasión, felicidad, alegría, empatía y compasión hacia el otro, así como mayor resiliencia que les ayude a disminuir los niveles de estrés y ansiedad durante la carrera, el ejercicio de su profesión y su vida personal.
- Favorecer el desarrollo del coraje para acercarse, comprender y relacionarse con el sufrimiento (propio y ajeno) sin tener que alejarse de él (es decir, prevenir el distrés empático), y el crecimiento de la motivación de aliviar el sufrimiento.
- Los participantes, estudiantes y futuros profesionales de la medicina, entrenados en compasión, al gozar de un mayor bienestar personal, tendrán una mayor capacidad de proporcionar una atención compasiva a sus pacientes, lo que redundará en proporcionarles un cuidado más eficaz, mejores resultados terapéuticos y una mejor convivencia de estos con su enfermedad, con el impacto positivo que esto supondrá en las familias.
- Establecer las bases en la Facultad de Medicina de la UCM para aportar a la sociedad médicos más compasivos y por ende, favorecer el crecimiento de una sanidad más humana, acorde con la tendencia a promover sistemas sanitarios centrados en el paciente
- Contribuir a que la Facultad de Medicina de la UCM sea un ejemplo de humanización de la enseñanza.

8.3. Información sobre el programa CCT™

El protocolo CCT™, desarrollado en la Universidad de Standford por académicos contemplativos, psicólogos clínicos e investigadores, y actualmente auspiciado por el Instituto de la Compasión. CCT™, es un programa secular que integra prácticas contemplativas tradicionales con la psicología contemporánea y la investigación científica sobre la compasión. El CCT™ es un curso semipresencial de 8 semanas que combina una sesión presencial de 2.30 horas de duración cada semana con la realización de prácticas durante la semana. El objetivo del CCT™ en medicina es desarrollar una

mente y un corazón compasivos que aporten habilidades para el auto-cuidado y fomenten una interacción compasiva con los pacientes.

La compasión, definida como sensibilidad hacia el sufrimiento propio y de los otros, junto con un compromiso profundo para tratar de aliviarlo, es una capacidad básica de cuidado inherente al ser humano que permite responder al sufrimiento con comprensión, paciencia y amabilidad, en lugar de con miedo y repulsión. La compasión puede entrenarse (Stepien, 2006; Hojat, 2009a, Goetz, 2010; Kelm, 2014), y mediante su práctica, mejorar la compasión por uno mismo y extenderla hacia aquellas personas que nos resultan difíciles. El programa CCT™ se basa en desarrollar una compasión genuina que surge de reconocer que el sufrimiento ajeno es semejante al propio y que al igual que nosotros, los otros también desean liberarse de dicho sufrimiento. De esta declaración, surge la preocupación empática que nos sensibiliza con el dolor de los otros. Lejos de implicar lástima (coloca a quien lo experimenta en una situación de superioridad), condescendencia, permisividad, o ausencia de límites, la compasión se relaciona con el coraje de enfrentarse al sufrimiento y permanecer frente a él, y con la motivación de hacer algo para disminuirlo.

Bibliografía

Stephen, K. A., & Baernstein, A. (2006). Educating for Empathy. J Gen Intern Med, 21(5).524–30. PMID: 16704404; PMC1484804. https://doi.org/10.1111/j.1525-1497.2006.00443.x

Hojat, M. (2009a). Ten approaches for enhancing empathy in health and human services cultures. J Health Hum Serv Adm, 31(4):412-450. PMID: 19385420.

Goetz, J. L., Keltner, D., & Simon-Thomas, E. (2010). Compassion: an evolutionary analysis and empirical review. Psychol Bull. 136(3), 351–374. PMID: 20438142 PMCID: 2864937 doi: 10.1037/a0018807

Kelm, Z., Womer, J., Walter, J. K., & Feudtner, C. (2014). Interventions to cultivate physician empathy: a systematic review. *BMC medical education*, *14*(1), 1-11. PMID: 25315848; PMCID: PMC4201694. https://doi.org/10.1186/1472-6920-14-219

Fotaki M. (2015). Why and how is compassion necessary to provide good quality healthcare? IntJ Health Policy Manag, 4(4), PMID: 25844380 PMCID: PMC4380560199–201. https://doi.org/10.15171/ijhpm.2015.66

Trzeciak, S., Roberts, B. W., & Mazzarelli, A. J. (2017). Compassionomics: Hypothesis and experimental approach. Med hypotheses, 107, 92-97. PMID: 28915973 doi: 10.1016/j.mehy.2017.08.015.

Kim, S. S., Kaplowitz, S., & Johnston, M. V. (2004). The effects of physician empathy on patient satisfaction and compliance. Eval Health Prof, 27(3), 237-251. PMID: 15312283 https://doi.org/10.1177/0163278704267037

Rakel, D. P., Hoeft, T. J., Barrett, B. P., Chewning, B. A., Craig, B. M., & Niu, M. (2009). Practitioner empathy and the duration of the common cold. Fam Med, 41(7), 494–501. PMID: 19582635; PMC2720820.

Hojat, M., Louis, D. Z., Markham, F. W., Wender, R., Rabinowitz, C., & Gonnella, J. S. (2011). Physicians' empathy and clinical outcomes for diabetic patients. Acad Medi, 86(3), 359-364. PMID: 21248604 doi: 10.1097/ACM.0b013e3182086fe1

Attar, H. S., & Chandramani, S. (2012). Impact of physician empathy on migraine disability and migraineur compliance. Ann Indian Acad Neurol, 15(Suppl 1), S89–94. PMID: 23024571; PMCID: PMC3444220 https://doi.org/10.4103/0972-2327.100025.

Del Canale, S., Louis, D. Z., Maio, V., Wang, X., Rossi, G., Hojat, M., & Gonnella, J. S. (2012). The relationship between physician empathy and disease complications: an empirical study of primary care physicians and their diabetic patients in Parma, Italy. Acad Med, 87(9), 1243-1249. PMID: 22836852 doi: 10.1097/ACM.0b013e3182628fbf

Steinhausen, S., Ommen, O., Antoine, S. L., Koehler, T., Pfaff, H., & Neugebauer, E. (2014). Short-and long-term subjective medical treatment outcome of trauma surgery patients: the importance of physician empathy. Patient Prefer Adherence, 8, 1239-53. PMID: 25258518; PMCID: PMC4173813. https://doi.org/10.2147/PPA.S62925

Moss, J., Roberts, M. B., Shea, L., Jones, C. W., Kilgannon, H., Edmondson, D. E., & Roberts, B. W. (2019). Healthcare provider compassion is associated with lower PTSD symptoms among patients with life-threatening medical emergencies: a prospective cohort study. Intensive Care Med, 45(6), 815-822. PMID: 30911803. https://doi.org/10.1007/s00134-019-05601-5

Zolnierek, K. B. H., & DiMatteo, M. R. (2009). Physician communication and patient adherence to treatment: a meta-analysis. Medical care, 47(8), 826-34. PMID: 19584762 doi: 10.1097/MLR.0b013e31819a5acc.

Burns, D. D., & Nolen-Hoeksema, S. (1992). Therapeutic empathy and recovery from depression in cognitive-behavioral therapy: a structural equation model. J Consult Clin Psychol, 60(3), 441–9. PMID: 1619098. doi: 10.1037//0022-006x.60.3.441.

Zachariae, R., Pedersen, C. G., Jensen, A. B., Ehrnrooth, E., Rossen, P. B., & von der Maase, H. (2003). Association of perceived physician communication style with patient satisfaction, distress, cancer-related self-efficacy, and perceived control over the disease. Br J Cancer, 88(5), 658-665. PMID: 12618870; PMCID: PMC237635. https://doi.org/10.1038/sj.bjc.6600798

Neumann, M., Wirtz, M., Bollschweiler, E., Mercer, S. W., Warm, M., Wolf, J., & Pfaff, H. (2007). Determinants and patient-reported long-term outcomes of physician empathy in oncology: a structural equation modelling approach. Patient Educ Couns, 69(1-3), 63-75. PMID: 17851016. https://doi.org/10.1016/j.pec.2007.07.003.

Fogarty, L. A., Curbow, B. A., Wingard, J. R., McDonnell, K., & Somerfield, M. R. (1999). Can 40 seconds of compassion reduce patient anxiety? J Clin Oncol, 17(1), 371-371. PMID: 10458256. https://doi.org/10.1200/JCO.1999.17.1.371

Epstein, R. M., Franks, P., Shields, C. G., Meldrum, S. C., Miller, K. N., Campbell, T. L., & Fiscella, K. (2005). Patient-centered communication and diagnostic testing. Ann Fam Med, 3(5), 415-421. PMID: 16189057 PMCID: PMC1466928. doi: 10.1370/afm.348

Epstein, R. M., Hadee, T., Carroll, J., Meldrum, S. C., Lardner, J., & Shields, C. G. (2007). Could this be something serious?. Reassurance, Uncertainty, and Empathy in Response to Patients' Expressions of WorryJ Gen Intern Med, 22(12), 1731-1739. PMID: 17972141 PMCID: PMC2219845 doi: 10.1007/s11606-007-0416-9

Kelm, Z., Womer, J., Walter, J. K., & Feudtner, C. (2014). Interventions to cultivate physician empathy: a systematic review. BMC Medi Edu, 14, 219. PMID: 25315848 PMCID: PMC4201694https://doi.org/10.1186/1472-6920-14-219

Shanafelt, T. D., Bradley, K. A., Wipf, J. E., & Back, A. L. (2002). Burnout and self-reported patient care in an internal medicine residency program. Ann Intern Med, 136(5), 358–367. PMID: 11874308 https://doi.org/10.7326/0003-4819-136-5-200203050-00008

McHugh, M. D., Kutney-Lee, A., Cimiotti, J. P., Sloane, D. M., & Aiken, L. H. (2011). Nurses' widespread job dissatisfaction, burnout, and frustration with health benefits signal problems for patient care. Health aff (Project Hope), 30(2), 202–210. PMID: 21289340 PMCID: PMC3201822https://doi.org/10.1377/hlthaff.2010.0100

Dzeng E. (2016). Moral Distress Amongst Physician Trainees Regarding Futile Treatments. J Gen Intern Med, 31(8), 830. PMID: 26951284 PMCID: PMC4945553 https://doi.org/10.1007/s11606-016-3648-8

Figley, C. R. (1995). Compassion fatigue: Toward a new understanding of the costs of caring. In B. H. Stamm (Ed.), Secondary traumatic stress: Self-care issues for clinicians, researchers, and educators (p. 3–28). The Sidran Press

van Mol, M. M., Kompanje, E. J., Benoit, D. D., Bakker, J., & Nijkamp, M. D. (2015). The Prevalence of Compassion Fatigue and Burnout among Healthcare Professionals in Intensive Care Units: A Systematic Review. PloS one, 10(8), e0136955. PMID: 26322644 PMCID: PMC4554995 doi: 10.1371/journal.pone.0136955

Sprang, G., Clark, J. J., & Whitt-Woosley, A. (2007). Compassion fatigue, compassion satisfaction, and burnout: Factors impacting a professional's quality of life. Journal of Loss and Trauma, 12(3), 259-280.

West, C. P., Dyrbye, L. N., Erwin, P. J., & Shanafelt, T. D. (2016). Interventions to prevent and reduce physician burnout: a systematic review and meta-analysis. Lancet (London, England), 388(10057), 2272–2281. PMID: 27692469https://doi.org/10.1016/S0140-6736(16)31279-X

Lamm, C., Decety, J., & Singer, T. (2011). Meta-analytic evidence for common and distinct neural networks associated with directly experienced pain and empathy for pain. NeuroImage, 54(3), 2492–2502. PMID: 20946964 doi: 10.1016/j.neuroimage.2010.10.014.

Klimecki, O. M., Leiberg, S., Ricard, M., & Singer, T. (2014). Differential pattern of functional brain plasticity after compassion and empathy training. Soc Cogn Affect Neurosci, 9(6), 873–879. PMID: 23576808; PMCID: PMC4040103. https://doi.org/10.1093/scan/nst060

Weng HY, Fox AS, Shackman AJ, et al. Compassion training alters altruism and neural responses to suffering. Psychol Sci. 2013;24(7):1171-1180. PMID: 23696200 PMCID: PMC3713090 doi:10.1177/0956797612469537

Hojat, M., Mangione, S., Nasca, T. J., Rattner, S., Erdmann, J. B., Gonnella, J. S., & Magee, M. (2004). An empirical study of decline in empathy in medical school. Med Educ, 38(9), 934–941. PMID: 15327674 https://doi.org/10.1111/j.1365-2929.2004.01911.x

Bellini, L. M., & Shea, J. A. (2005). Mood change and empathy decline persist during three years of internal medicine training. Academic medicine : journal of the Association of American Medical Colleges, 80(2), 164–167. https://doi.org/10.1097/00001888-200502000-00013

Neumann, M., Edelhäuser, F., Tauschel, D., Fischer, M. R., Wirtz, M., Woopen, C., Haramati, A., & Scheffer, C. (2011). Empathy decline and its reasons: a systematic review of studies with medical students and residents. Acad Med, 86(8), 996–1009. PMID:1670661 https://doi.org/10.1097/ACM.0b013e318221e615

Hojat, M., Vergare, M. J., Maxwell, K., Brainard, G., Herrine, S. K., Isenberg, G. A., Veloski, J., & Gonnella, J. S. (2009b). The devil is in the third year: a longitudinal study of erosion of empathy in medical school. Acad Med, 84(9), 1182–1191. PMID: 19707055 https://doi.org/10.1097/ACM.0b013e3181b17e

Wilson, S. E., Prescott, J., & Becket, G. (2012). Empathy levels in first- and third-year students in health and non-health disciplines. Am J Pharm Educ, 76(2), 24. PMID: 22438596 PMCID: PMC3305933https://doi.org/10.5688/ajpe76224PMID: 22438596 PMCID: PMC3305933 doi:10.5688/ajpe76224

Brazeau, C. M., Shanafelt, T., Durning, S. J., Massie, F. S., Eacker, A., Moutier, C., Satele, D. V., Sloan, J. A., & Dyrbye, L. N. (2014). Distress among matriculating medical students relative to the general population. Acad Med, 89(11), 1520–1525. PMID: 25250752 doi:https://doi.org/10.1097/ACM.0000000000000482

Association of American Medical Colleges (AAMC). Learning objectives for medical student education guidelines for medical schools. https:// members. aamc. org/ eweb/ upload/ Learning Objectives for Medical Student Educ Report I.pdf. https://www.aamc.org/system/files/c/2/493604-umecurriculumdashboardresource.pdf (consultado el 10-7-2020)

Patel, S., Pelletier-Bui, A., Smith, S., Roberts, M. B., Kilgannon, H., Trzeciak, S., & Roberts, B. W. (2019). Curricula for empathy and compassion training in medical education: A systematic review. PloS one, 14(8), e0221412. PMID: 31437225 PMCID: PMC6705835 doi: 10.1371/journal.pone.0221412

Patel, S., Pelletier-Bui, A., Smith, S., Roberts, M. B., Kilgannon, H. J., Trzeciak, S., & Roberts, B. W. (2018). Curricula and methods for physician compassion training: protocol for a systematic review. BMJ open, 8(9), e024320. PMID: 30224405 PMCID: PMC6144316 https://doi.org/10.1136/bmjopen-2018-024320

Weingartner LA, Sawning S, Shaw MA, Klein JB. (2019). Compassion cultivation training promotes medical student wellness and enhanced clinical care. BMC Med Educ, 10;19(1):139. PMID: 31077192; PMCID: PMC6511143. DOI: 10.1186/s12909-019-1546-6

8.4. Hipótesis

1. El programa estandarizado de Entrenamiento en el Cultivo de la Compasión (CCT) producirá una mejora del bienestar emocional y una reducción del malestar psicológico (estrés, ansiedad, depresión) y del burnout de los estudiantes de medicina post-programa en comparación con un grupo control en lista de espera.

2. Dichos cambios se mantendrán a lo largo del tiempo a los dos y seis meses tras la finalización del programa.

3. Los cambios mencionados estarán operados por incrementos en la compasión hacia un mismo, la compasión hacia otros, la atención al momento presente y las habilidades de regulación emocional.

4. Las habilidades desarrolladas en el programa supondrán un factor protector para el manejo del estrés y el malestar psicológico producido por la pandemia de la COVID-19.

9. DISEÑO

Se realizará un ensayo aleatorizado, controlado, unicéntrico con un grupo control en lista de espera (grupos paralelos). Se realizará una aleatorización 1:1 (mediante la función pertinente en Excell), con un enmascaramiento simple ciego. El simple ciego se garantiza mediante la división de lastareas en el equipo de investigación, de forma que la persona encargada de la evaluación y análisis de datos no participa y es ciega al proceso de aleatorización y a la conformación de los grupos (PR). La IP del proyecto (BR) será la encargada de citar a los participantes y realizar la asignación de los grupos.

9.1 Diseño del estudio

Realizaremos un análisis de las respuestas de los participantes a un protocolo online conformado por diferentes medidas psicológicas validadas psicométricamente, y que serán cumplimentados por los participantes en diferentes momentos temporales (Fig. 1). Dichos cuestionarios serán utilizados como indicadores cuantitativos de los cambios psicológicos experimentados por los participantes en las principales outcomes y targets del programa CCT®

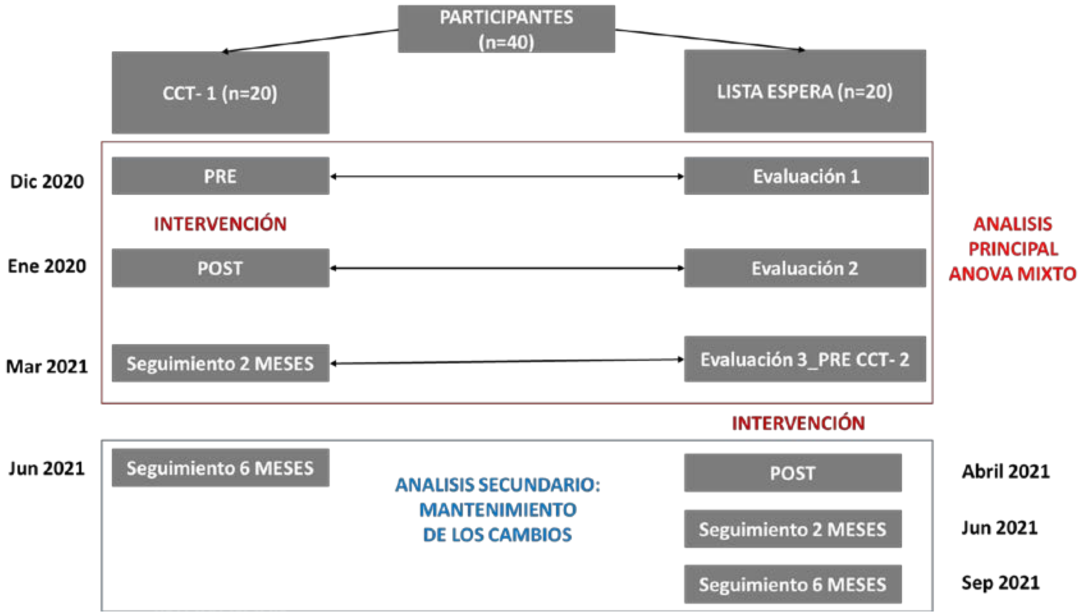

Figura 1. Diagrama CONSORT

En una **primera fase**, los participantes serán aleatorizados en los dos grupos de estudio (Fig. 1): grupo experimental (i.e., programa CCT) o grupo control en lista de espera. Dicha aleatroización se realizará tras la evaluación de línea base para evitar la aparición de sesgos producidos por las expectativas de participación en el programa CCT©.

En una **segunda fase**, a los 2 meses de haber finalizado el primer programa de CCT© (CCT-1), en febrero de 2021, se ofrecerá a los participantes en lista de espera la posibilidad de participar en el programa CCT© pasando a constituir el grupo CCT-2.

Las personas que voluntariamente quieran participar en la investigación se comprometerán a realizar una evaluación previa al programa CCT©, una breve evaluaciones inter-sesiones durante el programa, una evaluación al terminar el programa y una evaluación a los 2 y 6 meses de finalizado el mismo. Durante la primera fase del estudio, el grupo en lista de espera (grupo control) se someterá a las mismas evaluaciones y en los mismos tiempos que el grupo CCT-1.

9.2 Cuestionarios online

Serán aplicados mediante la plataforma online "Qualtrics". A continuación se especifican los detalles técnicos de los cuestionarios a cumplimentar por los participantes.

9.2.1 Bloque de cuestionarios pre-post programa (Tabla 1)

Tabla 1. Cuestionarios a cumplimentar por los participantes pre-post programa

Constructo	Instrumento
	Datos sociodemográficos y sanitarios*
	Experiencia previa en meditación
	Impacto del COVID-19 en los estudiantes
	**Mindfulness y compasión**
Mindfulness	Five Facet Mindfulness Questionnaire (FFMQ; Baer et al., 2006) → versión breve de 20 ítems
Compasión hacia uno mismo	Self-Compassion Scale (SCS-SF; Raes et al., 2011) → versión breve de 12 ítems
Compasión hacia otros	Escala de Compasión CS Pommier (CSP; Pommier et al., 2020) → 24 ítems
Empatía	Interpersonal Reactivity Index (IRI, Davis, 1980) → 14 ítems (sub-escalas de preocupación empática y malestar personal)
	**Psicopatología**
Estrés, ansiedad y depresión	Depression Anxiety Stress Scales (DASS – 21; Lovibond, & Lovibond, 1995) → 21 items.
Regulación emocional	Difficulties in Emotion Regulation Scale (DERS, Gratz & Roemer, 2004) → 28 items
Burnout	Maslach Burnout Inventory (version estudiantes) → 15 ítems
	**Bienestar**
Bienestar general	Pemberton Happiness Index (PHI, Hervas, & Vazquez, 2013) → 11 items
Resiliencia	Brief Resilience Scale (BRS; Smith et al., 2008) → 5 ítems

9.2.2 Bloque de cuestionarios inter-sesiones (una vez a la semana)

Las evaluaciones inter-sesiones estarán conformadas por un cuestionario breve dirigido a evaluar la adherencia al programa y la práctica semanal. Además, en el ecuador y al finalizar el programa, el participante cumplimentarán una encuesta anónima requerida por el Compassion Institute para evaluar la alianza con el programa, la alianza con el instructor y la satisfacción con el programa. Además, para garantizar y evaluar la adherencia al protocolo por parte del instructor, las sesiones del programa serán gravadas para su posterior revisión.

10. OBJETIVO PRINCIPAL

Objetivo principal

El objetivo de la presente propuesta es evaluar la eficacia y los cambios psicológicos producidos por un programa estandarizado de Entrenamiento en el Cultivo de la

Compasión (CCT©) en alumnos de medicina de la Universidad Complutense de Madrid en comparación con un grupo control en lista de espera.

Objetivos secundarios

1) Examinar si los cambios producidos por el programa se mantienen a los dos y seis meses tras la finalización del mismo.
2) Examinar los cambios en mecanismos psicológicos mediando los resultados positivos del programa. Evaluar si el entrenamiento en compasión es un factor protector frente al estrés y el malestar psicológico producido por la pandemia por COVID-19.

11. FÁRMACO EXPERIMENTAL Y CONTROL. DOSIS, FORMA FÍSICA, VÍA

11.1 Elección del programa CCT

El programa CCT© fue desarrollado en el año 2009 en la Universidad de Standford por académicos contemplativos, psicólogos clínicos e investigadores, y actualmente está auspiciado por el Compassion Institute. CCT© es un programa secular que integra prácticas contemplativas tradicionales con la psicología contemporánea y la investigación científica sobre la compasión. El CCT© es un curso semipresencial de 8 semanas que combina una sesión presencial de 2 horas de duración cada semana con la realización de prácticas durante la semana. El objetivo del CCT© en medicina es desarrollar una mente y un corazón compasivos que aporten habilidades para el auto-cuidado y fomenten una interacción compasiva con los pacientes.

La compasión, definida como sensibilidad hacia el sufrimiento propio y de los otros, junto con un compromiso profundo para tratar de aliviarlo, es una capacidad básica de cuidado inherente al ser humano que permite responder al sufrimiento con comprensión, paciencia y amabilidad, en lugar de con miedo y repulsión. La compasión puede entrenarse (Stepien, 2006; Hojat, 2009a, Goetz, 2010; Kelm, 2014), y mediante su práctica, mejorar la compasión por uno mismo y extenderla hacia aquellas personas que nos resultan difíciles. El programa CCT™ se basa en desarrollar una compasión genuina que surge de reconocer que el sufrimiento ajeno es semejante al propio y que al igual que nosotros, los otros también desean liberarse de dicho sufrimiento. De esta declaración, surge la preocupación empática que nos sensibiliza con el dolor de los otros. Lejos de implicar lástima (coloca a quien lo experimenta en una situación de superioridad), condescendencia, permisividad, o ausencia de límites, la compasión se relaciona con el coraje de enfrentarse al sufrimiento y permanecer frente a él, y con la motivación de hacer algo para disminuirlo.

11.2 Detalles del programa

El programa CCT© consta de 6 pasos que se desarrollan en ocho semanas. En concreto:

Semana 1. Asentamiento y enfoque
Desarrollo de atención plena (Mindfulness), asentar la mente en la experiencia presente con aceptación y sin juicio mediante ejercicios destinados a enfocar la atención en la respiración trayendo, con amabilidad, la atención de vuelta cada vez que cada vez que se distraiga.

Semana 2. La compasión como capacidad natural
Aprender a reconocer los signos físicos y psíquicos asociados a la compasión mediante el diálogo y los ejercicios guiados.

Semana 3. Autocompasión
Se presenta la idea de que el cultivo de la autocompasión es la base para desarrollar un comportamiento compasivo hacia los demás. Entrenamiento en la práctica del diálogo auto-compasivo, la auto-aceptación y crecimiento de estas habilidades en las situaciones difíciles.

Semana 4. Amor a uno mismo
Aprender a cultivar la calidez, el aprecio, la alegría y la gratitud por uno mismo.

Semana 5. La humanidad compartida
Reconocimiento de la humanidad compartida (ver al otro "tal como yo") e interdependencia como el fundamento de la compasión hacia los demás.

Semana 6. Compasión
A partir de la humanidad compartida y la interdependencia, cultivar de la compasión por todos los seres, incluidos los difíciles.

Semana 7. Compasión activa
Encaminada a desarrollar la estabilidad interna para acoger el sufrimiento de los demás y ofrecer la felicidad propia.

Semana 8. Práctica integrada de cultivo de la compasión
Congrega los elementos de los pasos anteriores en una práctica de meditación compasiva que puede seguirse como práctica diaria

Estos seis pasos, se desarrollarán mediante:

- 8 encuentros semanales de 2h de duración online (por motivos del COVID-19) consistentes en ejercicios relacionales, meditaciones y diálogos sobre la pedagogía y ciencia relacionados con el tema de la semana. Los encuentros online se llevarán a cabo a través de la plataforma Zoom.
- Meditaciones guiadas grabadas en audios de 20-30 min de duración para ser realizadas a diario (prácticas formales).

- Prácticas informales en el día a día

11.3 Procedimiento para evaluar el cumplimiento del participante

Las encuestas inter-sesiones junto al número de sesiones a las que asiste el participante se utilizarán para evaluar la adherencia al programa.

12. VARIABLE PRINCIPAL DE VALORACIÓN

- Variables principales:
  - Malestar psicológico: estrés, ansiedad, depresión.
  - Bienestar psicológico.
  - Burnout
  - Compasión
- Variables secundarias:
  - Mindfulness o atención al presente.
  - Empatía.
  - Regulación emocional.
  - Resiliencia.
  - Adherencia al programa.
  - Práctica diaria.

13. POBLACIÓN EN ESTUDIO Y NÚMERO TOTAL DE PACIENTES

13. 1. Método de cálculo de la muestra

El tamaño muestral se determinó a priori mediante el software G*Power (v. 3.0.10). El tamaño muestral requerido para analizar un ANOVA mixto (el factor grupo como medida independiente y el factor tiempo como medida relacionada), con un tamaño el efecto esperado de 0.40 (Brito-Pons et al., 2018), un nivel de significación de 0,05, y una potencia del 90%, el tamaño muestral estimado es de 44 participantes en toda la muestra (22 en cada grupo). Dicha estimación del tamaño muestral se ha llevado a cabo para el cambio pre-post en la variable de malestar emocional (i.e., estrés, ansiedad y depresión).

13.2. Estimación de la posible pérdida

Con el fin de reducir al máximo la pérdida muestral a lo largo del estudio, hemos adoptado diversas medidas dirigidas a mantener la motivación y reforzar la participación de los participantes: los integrantes del grupo control firmarán un acuerdo de realizar las evaluaciones pertinentes para formalizar la reserva de su plaza en la segunda promoción del curso. Una vez finalizado el estudio, se retribuirá a los participantes con

un crédito ECTS de libre configuración (aprobado por las Comisión de Estudios de la UCM de 12 de noviembre de 2020) y objeto de reconocimiento académico para el grado en medicina. Además, los participantes que terminen todas las evaluaciones del estudio recibirán un libro de regalo sobre la temática del programa. Por todo lo anterior, esperamos una pérdida muestral inferior al 5%.

13.4. Descripción del reclutamiento y requisitos de los participantes

El programa CCT® se ofertará a todos los alumnos de la facultad de medicina de la UCM matriculados en el curso 2020-21. Dicha oferta se realizará a través del Viderectorado de alumnos de la Facultad de Medicina de la UCM, de las pantallas de difusión de la biblioteca, de la asociación de alumnos de dicha facultad y delegados de curso. La selección de los participantes en el estudio se hará entre aquellos alumnos que soliciten la inscripción en el programa CCT®. En la selección, tendrán preferencia aquellos alumnos que estén en el 3º-6º año de formación por ser el periodo en el que están en contacto con los pacientes en los distintos hospitales asociados a la UCM.

Entre los criterios de inclusión del estudio se incluyen: 1) ser mayor de edad; 2) nivel suficiente de español para comprender las instrucciones y sesiones del programa; 3) estar matriculado en la facultad de medicina de la UCM durante el curso académico 2020-21; 4) compromiso de asistencia a la totalidad del programa; 5) otorgar el consentimiento informado para participar en el estudio; y 6) tener acceso a internet y ordenador para poder realizar las sesiones online.

Entre los criterios de exclusión se incluyen: 1) presentar un trastorno mental grave en fase activa; 2) estar bajo los efectos del alcohol u otras sustancias durante la evaluación de los criterios o las sesiones del programa; y 3) participar en otro programa estandarizado de meditación durante el trascurso del programa.

Se empleará el criterio de asistencia al 75% de las sesiones del programa como indicativo de “exposición mínima al tratamiento”.

14. ANÁLISIS ESTADÍSTICO

Siguiendo las recomendaciones CONSORT, se realizará Intention-To-Treat Analysis para el manejo de los datos perdidos. Se comenzará realizando un diagnóstico del patrón de aleatoriedad de la perdida mediante el test MCAR de Little. También se analizarán las diferencias en línea base entre completers (i.e., per-protocol) y missing para determinar si existe alguna variable en el conjunto de datos que predice la pérdida de datos. En función del patrón de pérdida se realizarán imputaciones múltiples o mediante algoritmos de Máxima Verosimilitud. Finalmente se realizarán análisis de sensibilidad comparando los datos per-protocol con los imputados para determinar si la imputación ha generado estimaciones sesgadas. Como se indica en el apartado anterior, se

empleará el criterio de asistencia al 75% de las sesiones del programa como indicador de "exposición mínima al tratamiento".

Se emplearan pruebas t de student y chi-cuadrado para analizar las diferencias entre grupos en la línea base. El análisis principal será un ANOVA mixto con dos factores: factor grupo de medidas independientes (experimental vs control) y factor tiempo de medidas repetidas (pre, post, seguimiento 2 y 6 meses). También se realizarán ANCOVAs usando la línea base como covariable en los análisis y ANOVAs de medidas repetidas para evaluar el mantenimiento de los cambios a lo largo del tiempo. Los análisis de varianza se complementaran con análisis mediadores y moderadores para estudiar los mecanismos de cambio del programa. Se definirá un nivel de significación de alfa 0,05 en los contrastes bilaterales. Los análisis estadísticos se llevarán a cabo mediante los softwares SPSS (v. 25) y R (v. 3.6.1).

15. CONSIDERACIONES ÉTICAS

Dada la naturaleza del estudio que se presenta a evaluación por el Comité Ético, el equipo de investigación seguirá en todo momento durante el desarrollo del ensayo las normas éticas marcada por el Código Deontológico del Psicólogo publicado por el Colegio Oficial de Psicólogos (https://www.cop.es/pdf/Codigo-Deontologico-Consejo-Adaptacion-Ley-Omnibus.pdf). Los participantes en la investigación no serán expuestos a ningún tipo de daño permanente, irreversible o innecesario, y su participación en la investigación será autorizada explícitamente mediante consentimiento informado (art. 34). Así mismo, en la investigación se respetará la dignidad de las personas, sus creencias, su intimidad y su pudor (art. 37). En cuanto al uso de la información, únicamente se recabará la información estrictamente necesaria para el desempeño de las tareas y siempre con la autorización expresa de los participantes en la investigación (art. 39).

Toda la información obtenida estará sujeta al deber y derecho de secreto profesional (art. 40) y será tratada con absoluta confidencialidad, manteniendo el anonimato de los participantes en cualquier publicación a la que el proyecto pudiera dar lugar. En cualquier caso, todos los integrantes de este proyecto se adecuarán a lo establecido en la Ley Orgánica 3/2018 del 5 de diciembre de Protección de Datos de Carácter Personal y al Real Decreto 1720/2007, de 21 de diciembre, que desarrolla dicha Ley.

El estudio que se propone a evaluación del Comité, supone un riesgo prácticamente inexistente para el participante ya que durante el desarrollo del programa, este será advertido reiteradamente a priorizar su bienestar frente a las prácticas y ejercicios recomendados así como a realizarlas dentro de una zona de seguridad emocional, siendo alentado a suspender las mismas y consultar al instructor ante cualquier incidencia. El instructor estará a disposición de los participantes durante todo la duración del estudio a través del correo electrónico, teléfono personal o

videoconferencia online para atender a las necesidades de los participantes. Además, durante todas las sesiones online y siguiendo las recomendaciones del Compassion Institute para ofrecer el programa CCT© online, y con el fin de la mejor monitorización de los participantes por parte del instructor durante las conexiones online, este estará preferiblemente auxiliado por otra persona en las cuestiones técnicas de la conexión. En el presente estudio, esta labor será realizada por una estudiante de medicina de sexto curso miembro del Proyecto de Innovación Docente en el que se enmarca el presente estudio.

El presente ensayo clínico utiliza cuestionarios y medidas ampliando utilizadas en la investigación clínica y psicológica (ver sección 9.2.1) y de la meditación (fundamentalmente aspectos atencionales y fenomenológicos). Todos los participantes son voluntarios y si se observa una puntuación elevada en los cuestionarios de depresión o ansiedad se les informará de la disponibilidad de servicios de asistencia, si así lo desean, tanto en la Facultad de Psicología de la UCM como en el ámbito del sistema público de salud. La participación en el equipo del proyecto de una Licenciada en Medicina y Cirugía (María Blanca Rojas López) y un graduado en Psicología (Pablo Roca Morales) garantizan el seguimiento adecuado de la evolución emocional de los participantes. Teniendo en cuenta el carácter exploratorio del estudio (se trata de uno de los primeros EC aleatorizado aplicando el programa CCT en futuros profesional médico), consideramos que un grupo control en lista de espera cumple los requisitos pertinentes para responder a las hipótesis del estudio.

El Instituto Nirakara (miembro de la Cátedra Extraordinaria de «Mindfulness y Ciencias Cognitivas» de la Universidad Complutense de Madrid) y participante en el presente ensayo clínico, observa de manera rigurosa los aspectos éticos y los procedimientos de manejo de datos, cumpliendo con la LOPD. Dicho Instituto ha pasado una auditoría de la Agencia de Protección de Datos teniendo sus ficheros dados de alta para fines de investigación de "grado alto". Específicamente la auditoría ha verificado y aprobado la norma del empleo de "muestras no asociadas a una persona identificada o identificable por haberse sustituido o desligado la información que identifica a esa persona utilizando un código que permita la operación inversa". Este procedimiento está contemplado en la propuesta presente.

La información a los participantes será proporcionada por la responsable del Proyecto de Innovación Docente en el que se inscribe este ensayo y que a su vez es la investigadora principal del ensayo clínica e instructora del programa CCT©.

Se permitirán las monitorizaciones, auditorias, revisiones del CEIC e inspecciones reguladoras relacionadas con el ensayo, facilitando el acceso directo a los documentos / datos originales.

16. DURACIÓN DEL TRATAMIENTO (de la permanencia en el EC en este caso)

Los participantes permanecerán formalmente dentro del ensayo clínico desde noviembre de 2020 hasta junio y octubre de 2021 según que pertenezca al grupo CCT-1 o CCT-2 respectivamente.

17. CALENDARIO Y FECHA PREVISTA DE FINALIZACIÓN

Inicio en la primera quincena de noviembre de 2020 y finalización en la segunda quincena de septiembre de 2021.

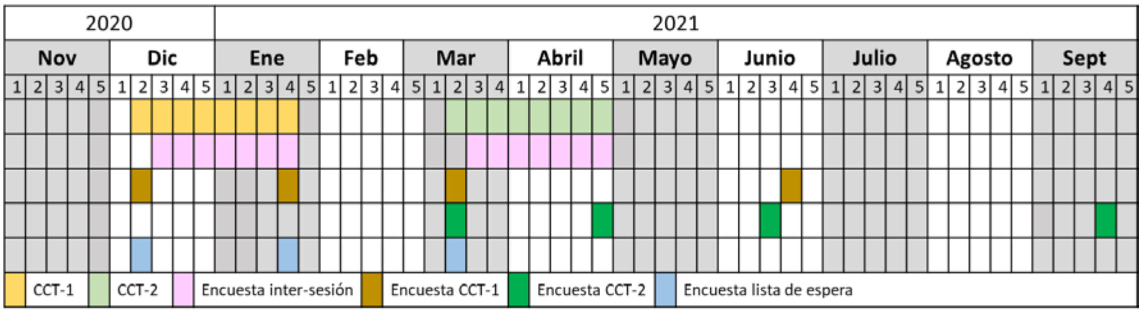

Supplement: S4 File — (PDF) [file pone.0287388.s005.pdf]
