# Supplementary material for: A compassion-based program to reduce psychological distress in medical students: A pilot randomized clinical trial
Source: PLoS One. 2023 Jun 23;18(6):e0287388. doi: 10.1371/journal.pone.0287388 (PMC10289411; doi:10.1371/journal.pone.0287388)
Supplement: Attachment — Submitted filename: renamed_45aaa.docx [file pone.0287388.s006.docx]

# COMMENTS – Manuscript PONE-D-23-02561

Title: “A compassion-based program to reduce psychological distress in medical students: a pilot randomized clinical trial”

**Important note: This review pertains only to ‘statistical aspects’ of the study and so ‘clinical aspects’ [like medical importance, relevance of the study, ‘clinical significance and implication(s)’ of the whole study, etc.] are to be evaluated [should be assessed] separately/independently. Further please note that any ‘statistical review’ is generally done under the assumption that (such) study specific methodological [as well as execution] issues are perfectly taken care of by the investigator(s). This review is not an exception to that and so does not cover clinical aspects {however, seldom comments are made only if those issues are intimately / scientifically related & intermingle with ‘statistical aspects’ of the study}. Agreed that ‘statistical methods’ are used as just tools here, however, they are vital part of methodology [and so should be given due importance]. I look at the manuscript in/with statistical view point, other reviewer(s) look(s) at it with different angle so that in totality the review is very comprehensive. However, there should be efforts from authors side to improve (may be by taking clues from reviewer’s comments). Therefore, please do not limit the revision only (with respect) to comments made here.**

**COMMENTS:** I noted (i.e., in my opinion) that your ABSTRACT is well drafted, but is ‘assay type’. It is preferable [refer to item 1b of CONSORT checklist 2010: Structured summary of trial design, methods, results, and conclusions] to divide the ABSTRACT with small sections like ‘Objective(s)’, ‘Methods’, ‘Results’, ‘Conclusions’, etc. which is an accepted practice of most of the good/standard journals [including this one, though ‘The PLoS One Guidelines to Authors’ did not specify an Abstract format, it is desirable]. It will definitely be more informative then, I guess, whatever the article type may be [including a pilot randomized clinical trial].

A very small correction: meaning/longform of CCT [Compassion Cultivation Training] is given in the ‘abstract’ itself but on second time use of the term, whereas, it is a general convention to give it at first time use of the term. Of course, it a very minor correction suggested. It is stated in ‘abstract’ that “This pilot study aims to examine the effectiveness of Compassion-Based Interventions (CBIs) in preventing psychological distress” [is not ‘Interventions’ plural?] but next sentence “We hypothesize that the CCT program, as compared to a waitlist control group” indicates that this study deals with only one intervention namely CCT program. Is not that so? It is pardonable if it is by oversight/typing mistake, however, please clarify.

It seems that this ‘Clinical Trial’ is not register as desired. If it is, then why the number {ClinicalTrial.org (ID: XXX)} is not specified? This study being ‘pilot’ in nature, sample size is not a big issue. However, [though many things are ignored (loosely looked at / evaluated)] in case of ‘pilot studies’, methodological issues need to be very rigorous followed {like in case of clinical trial, CONSORT guidelines are to be strictly observed/followed}. Then the term ‘CONSORT’ is a very vital term [but surprisingly it appears only once in ‘Strengths, limitations, and future directions’ section]. Even important items of/in CONSORT checklist are not found [since your article type is ‘Clinical Trial’, you are supposed to cover these items in the report (& note that CONSORT for Pilot trial is/are also available).

According to document on CONSORT for Pilot trial “Formal hypothesis testing for effectiveness (or efficacy) is not recommended. The aim of a pilot trial is not to assess effectiveness (or efficacy) and it will usually be underpowered to do this” (check your aim stated). In addition, please note that any regression techniques [including Mixed-effects models which are conducted here to analyze the effects of the CCT program] are not basically/originally developed for any sort of [between or within group(s)] comparison(s). Using ‘Mixed-effects models’ is definitely not wrong; however, some head-to-head comparison is expected. Further, it may be noted that

Though the measures/tools used are appropriate [refer table-2], most of them are likely to yield data that are in ‘ordinal’ level of measurement [and not in ratio level of measurement for sure {as the score two times higher does not indicate presence of that parameter/phenomenon as double (for example, a Visual Analogue Scales VAS score or say ‘depression’ score)}]. Then application of suitable non-parametric test(s) is/are indicated/advisable [even if distribution may be ‘Gaussian’ (also called ‘normal’)]. Agreed that there is/are no non-parametric test(s)/technique(s) available to be used as alternative in all situation(s) [suitable / most desired/applicable], but should be used whenever/wherever they are available. Therefore, in short use suitable non-parametric test(s)/technique(s) while dealing with data that are in ‘ordinal’ level of measurement even if [despite that] the distribution may be ‘Gaussian’. Testing ‘normality’ in sample [by using any normality test(s)} is not required/desired while dealing with data that are in ‘ordinal’ level of measurement [as most of the normality tests are not valid for ‘ordinal’ data].

I request authors to check contents of references 77,78. Because in my knowledge, at least article 77 concludes as follows:

**Conclusions:** Engagement in MBSR is not predictive of increased rates of harm relative to no treatment. Rather, MBSR may be protective against multiple indices of harm. Research characterizing the relatively small proportion of MBSR participants that experience harm remains important.

Moreover, it may please be noted that “Absence of evidence is not evidence of absence” [Altman DG, Bland JM. BMJ volume 311, 1995, p 485 (Reprinted: Australian Veterinary Journal 1996;74, 311)]. {Even when P-value is not significantly lower that is null hypothesis of no difference / no association is not rejected, (in short, result is not significant), that does not amount to evidence of absence i.e., it does not imply that there no difference / no association. It only implies that there is no (i.e., these samples do not provide) [say enough] evidence to prove (rather indicate with certain specified confidence level) the difference / association}. Therefore, conclusion(s) from any study [in which result(s) is/are not significant], should be drawn in the light of this fact. Particularly look at (due consideration of) the hypothesis in each of the study quoted in reference 78.

Although the ‘Intervention description’ is indeed very good and useful, [as pointed out in ‘important note’ above] note that “This review pertains only to ‘statistical aspects’ of the study and so ‘clinical aspects’ should be assessed separately/independently [one should carefully consider/look at the clinical implications of the study].

In my opinion, to rescue this article (which is quite possible and easy), a small amount of re-vision (re-drafting) may be needed. However, please do not limit the revision only (with respect) to comments made here. More improvement is expected. Recommending minor revision.
