# Supplementary material for: A compassion-based program to reduce psychological distress in medical students: A pilot randomized clinical trial
Source: PLoS One. 2023 Jun 23;18(6):e0287388. doi: 10.1371/journal.pone.0287388 (PMC10289411; doi:10.1371/journal.pone.0287388)
Supplement: Attachment — Submitted filename: ref.docx [file pone.0287388.s007.docx]

“A compassion-based program to reduce psychological distress in medical students: a

pilot randomized clinical trial”

The present study aims to investigate the impact of Compassion Cultivation Training on psychological outcomes in a sample of medical students. The topic is exciting, and the contribution that the current study could provide to scientific literature is influential and essential.

Following this, I reported a series of suggestions to help authors improve the manuscript.

Background:

Since there are other types of Protocol Intervention, CBT-oriented focused on increases in

“Compassion, empathy and mindfulness skills, reductions of

psychological distress (stress, anxiety, and depression) and burnout, improvements in

well-being and emotion regulation, among others” I suggest that authors explain better why they chose this specific training and not others.

Materials and Methods:

The number of the study protocol approved by the Ethical Committee and the pre-registration at ClinicalTrial.org need to be included.

Participants:

I kindly ask the authors to specify the background related to the decision to set a medium effect size to estimate sample size, also considering that this field is novel and not extensively investigated, it should be expected to have a sample composed of a higher number of participants to extrapolate information about efficacy in a pilot study.

Discussion:

Since the final sample comprised fewer participants than expected, I suggest that authors better explain the implication of this fact on the outcome interpretation. I suggest adding this part to the conclusion section.
